# Plant-mediated rifampicin treatment of *Bemisia tabaci* disrupts but does not eliminate endosymbionts

**DOI:** 10.1038/s41598-022-24788-0

**Published:** 2022-12-01

**Authors:** Milan Milenovic, Antoine Gouttepifre, Michael Eickermann, Jürgen Junk, Carmelo Rapisarda

**Affiliations:** 1 Environmental Research and Innovation Department (ERIN), Luxembourg Institute of Science and Technology (LIST), 41, Rue du Brill, 4422 Belvaux, Luxembourg; 2 Dipartimento di Agricoltura, Alimentazione e Ambiente (Di3A), Università Degli Studi di Catania, Via Santa Sofia 100, 95123 Catania, Italy; 3 ESA, L’École Supérieure Des Agricultures, 55, Rue Rabelais, B.P. 30748, 49007 Angers Cedex 01, France

**Keywords:** Entomology, Symbiosis, Antibiotics

## Abstract

Whiteflies are among the most important global insect pests in agriculture; their sustainable control has proven challenging and new methods are needed. Bacterial symbionts of whiteflies are poorly understood potential target of novel whitefly control methods. Whiteflies harbour an obligatory bacterium, *Candidatus* Portiera aleyrodidarum, and a diverse set of facultative bacterial endosymbionts. Function of facultative microbial community is poorly understood largely due to the difficulty in their selective elimination without removal of the primary endosymbiont. Since the discovery of secondary endosymbionts, antibiotic rifampicin has emerged as the most used tool for their manipulation. Its effectiveness is however much less clear, with contrasting reports on its effects on the endosymbiont community. The present study builds upon most recent method of rifampicin application in whiteflies and evaluates its ability to eliminate obligatory Portiera and two facultative endosymbionts (Rickettsia and Arsenophnus). Our results show that rifampicin reduces but does not eliminate any of the three endosymbionts. Additionally, rifampicin causes direct negative effect on whiteflies, likely by disrupting mitochondria. Taken together, results signify the end of a rifampicin era in whitefly endosymbiont studies. Finally, we propose refinement of current quantification and data analysis methods which yields additional insights in cellular metabolic scaling.

## Introduction

Whiteflies, especially those of the *Bemisia tabaci* species complex, are some of the most important insect pests worldwide due to their invasiveness and transmission of plant viruses causing hefty economic losses^1,2^. At the same time, management of whiteflies and whitefly borne viruses is challenging due to their ability to attain insecticide resistance or efficient virus transmission before control methods show effects^3,4^. Diverse strategies are being investigated for more effective and environment friendly whitefly management methods, one of which is trying to exploit bacterial endosymbionts of whiteflies^5^. This idea is as old as the discovery of the critical role that endosymbionts play in enabling phloem feeders to survive on a very imbalanced diet^6^. In a bigger picture, whitefly holobiont is one of the most diverse among all insects^7^. Untangling the interactions between the organisms would deepen our understanding of microbial communities which through complex, context-dependent interactions with their host allow colonization of a unique ecological niche and provide adaptive plasticity in changing environments^8^.

The whitefly (*B. tabaci*) holobiont is one of the most diverse among all insects. *B. tabaci* harbours an obligatory bacterial endosymbiont alongside being host to zero to multiple facultative endosymbionts. The obligatory endosymbiont Candidatus Portiera aleyrodidarum synthesize and complement essential amino acids, vitamins and carotenoids that are lacking in the whitefly genome repertoire and are also deficient in the phloem sap^9,10^. Prevalence and diversity of bacterial secondary endosymbionts belonging to the genera *Arsenophonus, Cardinium, Fritschea, Hamiltonella, Hemipteriphilus, Rickettsia*, and *Wolbachia* vary across the different *B. tabaci* species and are not congruent with the phylogenetics of their whitefly host^11^. Reports of new facultative endosymbionts within *B. tabaci* have been rare in recent years indicating that the genetic diversity has been well explored. Roles of these facultative endosymbionts are however much less clear. They provide evolutionary advantage in certain environmental conditions while being neutral or detrimental in others, as for example it is evidenced by dramatic shifts of Rickettsia prevalence in *B. tabaci* populations over time reported by Cass, et al.^12^. Therefore, they can together be described as context-dependent modifiers of whitefly biology. Their presence and composition in whitefly populations varies over time and space in a so far largely unexplained manner^5^. Understanding the roles of facultative endosymbionts is essential in predicting how whiteflies will behave in new environments such as with changing climate and how we can manipulate them for the control of whiteflies and whitefly borne viruses.

Studies of endosymbiont roles have been hindered by the difficulty in obtaining whitefly populations with different endosymbiont compositions^5^. Facultative endosymbionts are especially difficult to study due to their conditional effects on the biology^13,14^. The environment and whitefly genome simultaneously affect both endosymbiont composition and whitefly biology directly. Separating the effects of whitefly genetic background, the environment, and endosymbiont community on the overall whitefly behaviour and fitness is challenging and necessitates whitefly populations with identical background but different endosymbiont composition. The main method explored so far for selective elimination of whitefly endosymbionts, and for probing the effects of modified endosymbiont densities, is the use of antibiotics. There are over a dozen published studies involving antibiotics and whitefly endosymbionts^15–28^. The antibiotics were used for two purposes: to either attempt to selectively eliminate secondary endosymbionts, or to temporarily reduce their densities with the hope of observing the effects which lower densities have on the insect biology.

The two most common methods of elimination of symbionts are the delivery of antibiotics supplemented in water sucrose solution through parafilm-based artificial feeder or the delivery through the plant phloem. Recently, a selective and stable elimination of whitefly endosymbionts have been claimed using the phloem delivery method, although other studies report mixed results^15^. Nevertheless, a move from delivery of antibiotics in water sucrose solution to delivery through the plant phloem seems to result in more efficient and precise manipulation of the endosymbiont community. This is hypothesized to be the case because plant phloem delivery method is able to deliver antibiotics over longer periods of time, alongside natural diet of whiteflies.

Several antibiotics have been tested on whitefly endosymbionts so far, with their selection mostly being guided by the patterns of antibiotic sensitivity in distantly related culturable bacteria^23^. Tested antibiotics include rifampicin, ampicillin (ampicillin trihydrate), tetracycline (oxytetracycline hydrochloride), chloramphenicol, penicillin, and antimicrobial enzyme lysozyme. Over time, rifampicin emerged as the most potent and widely used, with majority of the studies using only rifampicin^15–28^. Published studies reveal the pattern of poor selectivity of these antibiotics between different endosymbiont species present in whiteflies, although there are definitive differences in their sensitivity to antibiotics^15^. Major reduction of facultative endosymbionts without substantial reduction of the primary endosymbiont is also not possible. Differential response between the obligatory Portiera and a secondary endosymbiont was only reported for Hamiltonella which showed faster reduction rates, resulting in a possibility of eliminating Hamiltonella without eliminating Portiera^15^. Successful elimination of Hamiltonella using rifampicin was also reported using the artificial diet method by Su, et al.^29^. Rickettsia and Cardinium show similar response to rifampicin as Portiera, while use of tetracycline resulted in higher reduction of Cardinium compared to other endosymbionts^15^. Rate of reduction of Wolbachia and Arsenophonus in comparison to Portiera were not robustly tested to date. Other secondary endosymbionts, Fritschea and Hemipteriphilus, are rare in whiteflies and have not been included in antibiotic experiments so far.

The direct effect of antibiotics on whiteflies is another major problem with their use in the studies of bacterial endosymbionts. Negative effects have long been speculated but never demonstrated to date^16,19^. For example, the binding site of rifampicin is the bacterial DNA-dependent RNA polymerase^30^, but ability to bind to mitochondrial RNA polymerase in eukaryotes has been demonstrated in the rat liver cells^31^. The direct negative effects of rifampicin in whiteflies could limit its usability in studying roles of endosymbionts and their effects on whitefly biology as it is impossible to distinguish them from the effects of reduced endosymbiont densities.

The present study aims to re-evaluate and quantify the effects of rifampicin on the obligatory Portiera, and two facultative endosymbionts, Rickettsia and Arsenophonus, using the latest antibiotic delivery method. We advance the method of whitefly endosymbiont density quantification by introducing a normalization method, with the aim of reducing the measurement variability and to probe the relationship between nuclear DNA (nDNA) content, mitochondrial DNA (mtDNA) content, and endosymbiont densities. Further, we evaluate the feasibility of selective elimination of these facultative endosymbionts and attempt to pinpoint the cause of the challenges. Finally, we test the hypothesis that whitefly mitochondria are negatively affected by rifampicin and ask the question if antibiotic experiments can still yield useful insights into whitefly-endosymbiont interactions.

## Materials and methods

### Plant material

For whitefly rearing and antibiotic experiments, tomato (*Solanum lycopersicum* L.) plants of the cultivar ‘Moneymaker’ were grown from seed (Kiepenkerl, Bruno Nebelung GmbH, Everswinkel, Germany) in an insect-proof cage (50 × 50 × 50 cm) at 22 °C, 50% RH and photoperiod of 12L:12D under Valoya C65 lights with NS12 spectrum (Valoya, Helsinki, Finland). Plants potted in 5 l pots plants were fertilized weekly by irrigating with 200 ml of Peters Professional Allrounder (Dublin, OH, United States) water soluble fertilizer at the concentration of 2 g/l.

### Insect colony

The laboratory whitefly colony was established by introducing eggplant leaves infested with pupal stage of *B. tabaci* MED to the insect proof cage containing a tomato plant. The infested leaves were collected in October 2019, from eggplant grown in a greenhouse in south-eastern Sicily (Vittoria, province of Ragusa, Italy; 36.97134° N, 14.424505° E). The petioles of leaves were kept in water in the cage for 48 h to allow eclosion of whitefly adults. Established colony was maintained on tomato for over ten generations before being used for any experiment. Colony was maintained by regularly transferring whitefly adults to new tomato plants.

### Whitefly identification

Whitefly DNA was extracted and PCR was performed as described in Milenovic, et al.^32^. In short, DNA was extracted from ten whiteflies individually, and for each a fragment of mitochondrial Cytochrome Oxidase I gene (mtCOI) was amplified using Q5® Hot Start High-Fidelity 2X Master Mix (New England Biolabs, MA, United States), and sanger sequenced. Obtained sequences were subsequently aligned with the mtCOI reference dataset as published by Boykin, et al.^33^ using MAFFT v7.490^34^ with FFT-NS-I method and manually inspected for potential errors and trimmed to the same length (657 bp). The most suitable evolution model was determined to be GTR + I + G using MrModeltest v2.4^35^. Selected model was used to perform Bayesian inference phylogenetic analysis using MrBayes v3.2.7a^36–38^. Two independent runs of Markov Chain Monte Carlo (MCMC) were performed with 64 chains and sampling/diagnostic frequency of 1000 until average standard deviation of split frequencies fell below 0.01, which happened after 28,087,000 generations. Bayesian posterior probabilities were subsequently calculated and are presented for each node of the phylogenetic tree. The burn-in fraction was set to 25% for MCMC, SUMP and SUMT commands. The resulting tree was drawn using FigTree v1.4.4^39^. The process was repeated once again, and the resulting tree was compared to the first run pair to confirm that the analysis was not trapped in the local optima. Whitefly biotype was then identified.

### Endosymbiont identification

Whitefly endosymbionts were detected and identified by means of PCR amplification and sequencing of a portion of 16S rDNA gene of each endosymbiont described in whiteflies to date. Amplification was performed using Q5® Hot Start High-Fidelity 2X Master Mix (New England Biolabs, MA, United States). A PCR consisted of 30 s initial denaturation at 98 °C, followed by 35 cycles of 7 s denaturation at 98 °C, primer annealing for 20 s and extension for 20 s at 72 °C, followed by final extension of 2 min at 72 °C. Each endosymbiont was targeted using primer set at the optimal annealing temperature as described in Table 1. Amplicons from positive reactions were sequenced using Sanger sequencing method. Raw sequencing reads were manually trimmed to remove low quality bases. Forward and reverse sequences were aligned and assembled using CLC Main Workbench v21.0.1 (QIAGEN, Aarhus, Denmark). Sequences were identified by performing BLAST search at NCBI (http://www.ncbi.nlm.nih.gov) using default settings. In case of Portiera and Rickettsia, BLAST search was enough to identify the phylogenetic group as there was 100% sequence identity with sequences previously subjected to phylogenetic analysis by Kanakala and Ghanim^11^. Arsenophonus sequence did not yield identical match. To determine the phylogenetic placement, Arsenophonus sequences from the study of Kanakala and Ghanim^11^ were downloaded, as well as 10 other top BLAST hits, and phylogenetic analysis was performed using the same method as for the identification of whitefly biotype described above. Multiple sequence alignment was trimmed to the same length (616 bp). The best model was identified to be HKY + G.

**Table 1:** PCR primers and corresponding annealing temperatures used for whitefly and endosymbiont detection and identification.

Organism	Target	Oligo ID	Fragment (bp)	Ta (°C)	Sequence 5’-3’	Source
Whiteflies	COX1	Bt-MM1-F	1300	56	CTGAYATRGCKTTTCCTCG	Present study
		Bt-MM1-R			TTACTGCAYWTTCTGCCAC	
Portiera	16S rRNA	Port-F	900	56	GGAAACGTACGCTAATAC	Thierry, et al.^52^
		Port-R			TGACGACAGCCATGCAGCAC	
Arsenophonus	23S rRNA	Ars23S-F	600	60	CGTTTGATGAATTCATAGTCAAA	Thao and Baumann^53^
		Ars23S-R			GGTCCTCCAGTTAGTGTTACCCAAC	
Cardinium	16S rRNA	CLO-F	400	61	GCGGTGTAAAATGAGCGTG	Weeks, et al.^54^
		CLO-R			ACCTMTTCTTAACTCAAGCCT	
Fritschea	16S rRNA	U23-F	600	69	GATGCCTTGGCATTGATAGGCGATGAAGGA	Everett, et al.^55^
		23SIG-R			TGGCTCATCATGCAAAAGGCA	
Hamiltonella	16S rRNA	Ham-F	750	65	TGAGTAAAGTCTGGGAATCTGG	Zchori-Fein and Brown^56^
		Ham-R			AGTTCAAGACCGCAACCTC	
Rickettsia	16S rRNA	Rb-F	960	61	GCTCAGAACGAACGCTATC	Chiel, et al.^57^
		Rb-R			GAAGGAAAGCATCTCTGC	
Wolbachia	16S rRNA	V1	900	55	TTGTAGCCTGCTATGGTATAACT	O'Neill, et al.^58^
		V6			GAATAGGTATGATTTTCATGT	

### Endosymbiont localization

Localization of detected endosymbionts was performed using fluorescence in situ hybridization technique (FISH), using a modified protocol of Gottlieb, et al.^40^. Whitefly adults and nymphs were collected from the plant and immediately fixed in Carnoy’s fixative (6:3:1 mixture of chloroform, ethanol and glacial acetic acid) overnight. Whitefly eggs were collected and fixed together with a small piece leaf tissue to which the eggs were attached, which allowed easier manipulation of the sample. Fixation was followed by thorough wash in 100% ethanol and overnight decolourization in 6% alcoholic H_2_O_2_ solution. Specimens were washed again three times in 100% ethanol, and 3 times in PBST solution (1X PBS with 0.3% Triton X100). Next, specimens were washed 3 times in probe-free hybridization buffer (20 mM Tris–HCl pH 8.0, 0.9 M NaCl, 0.01% sodium dodecyl sulfate, 30% formamide, PCR-grade H_2_O) and incubated in the same buffer for 15 min. Buffer was then replaced with the same buffer supplemented with FISH probes (5’ Cy3-TGTCAGTGTCAGCCCAGAAG 3’ and 5’ Cy5-TCCACGTCGCCGTCTTGC 3’ for Portiera and Rickettsia, respectively) at 100 pmol/ml concentration and hybridized overnight in the dark at 22 °C. To reliably detect Arsenophonus which is present in lower densities, a doubly labelled FISH probe (5’ Cy3-TATCGCAGGAGAAAAGTCTG-Cy3 3’) was used with the same hybridization protocol as for the other endosymbionts^41^. Post hybridization wash was carried out by washing 3 times with probe-free hybridization buffer this time supplemented with 0.1 mg/ml DAPI as a DNA counterstain. All steps were performed in 200 µl sterile PCR tubes. Whiteflies were then mounted using hard setting VECTASHIELD® Vibrance™ Antifade Mounting Media (Vector Laboratories, Inc., United States) and observed under Zeiss LSM 880 confocal laser scanning microscope. The same protocol was followed for whitefly eggs, nymphs and adults. In total 5 eggs, 7 nymphs and 9 adults were imaged. Finally, as an independent supplement to qPCR quantification, nine antibiotic treated individuals of different developmental stages were also subjected to FISH followed by laser scanning confocal microscopy in the same way.

### Antibiotic treatment

Plants used to deliver antibiotic solution were established by rooting tomato side shoot cuttings with five fully developed leaves in an insect-proof cage (50 × 50 × 50 cm), kept at the same conditions as the whitefly colonies. Side shoots were cut and immersed into an opaque 250 ml glass bottle containing water solution with 1 g/l Peters Professional Allrounder fertilizer (ICL Specialty Fertilizers, Ohio, USA) and kept until extensive root system developed. A total of six plants were established. At this point, about 50 unsexed whitefly individuals per plant were introduced to the cage. Whitefly adults were subsequently removed after one week. When the whitefly eggs were close to hatching, water-fertilizer solution in five out of six plants was replaced with water solution containing 25 mg/l of rifampicin antibiotic (AppliChem GmbH, Darmstadt, Germany). Water-fertilizer solution of the sixth plant was replaced with water to serve as a negative control and moved to a different cage. During the experiment, the loss of solution due to uptake by the plant and evaporation was replenished daily by adding water. After 10 days, the solution was replaced entirely with fresh antibiotic solution, or watery for the treated and control plants respectively. The treatment continued until the eclosion of adults. At this point, whitefly adults were collected by aspiration, sexed, and individually stored in 200 µl of ethanol until DNA extraction. Additional adults were collected in the same way from the laboratory population, not subject to any experiments.

### Endosymbiont quantification

DNA extraction was performed the same way as for the whitefly identification described above. Number of analysed individuals was 48 females and 48 males in the antibiotic treated group, 24 females and 16 males in negative control group, and 24 females and 24 males in the rearing colony group. Relative quantity of whitefly endosymbionts was determined using TaqMan probe method of quantitative polymerase chain reaction (qPCR) using Takyon™ Low ROX Probe 2X MasterMix dTTP blue reagents (Eurogentec, Seraing, Belgium). Multiplex primer/probe sets were designed for Portiera and Rickettsia, one set for Arsenophonus, and two additional primer/probe sets were designed targeting whitefly nuclear DNA (portion of Actin gene), and mitochondrial DNA (portion of COX1 gene) to enable normalization and to investigate the effects of rifampicin on whitefly mitochondria. Whitefly nuclear DNA primers were design based on the α-actin NCBI GenBank sequences KJ913697.1, KC161211.1 and MN738077.1. Mitochondrial DNA, Portiera, and Rickettsia primers and probes were designed based on NCBI GenBank sequences MH205753, CP003835.1 and CP016305.1 respectively. Arsenophonus primers and probes were designed based on the alignment of all available Arsenophonus sequences with at this time still unpublished Arsenophonus genome sequencing reads. Probes were purified using RP-HPLC method and primers desalted. All qPCR primers and probes used in this study are presented in Table 2. Standard curves for each target were constructed based on 10-step 1:2 serial dilution of a sample with highest DNA concentration as determined by spectrophotometer, with three replicates per point. As the total number of samples was 184, the samples needed to be split between two 96-well plates. To account for any variability between runs, each treatment group was equally represented in both plates. As an additional check, due to the impracticality of running all 10-point serial dilution samples on all plates for all targets, the consistency between runs was verified by including simplified 3-point dilution of the same sample on all plates. Finally, a negative control sample was included in all plates. The assay was performed on ViiA 7 Real-Time PCR instrument (Thermo Fisher Scientific, Massachusetts, USA). Threshold cycle (Ct) values were determined using QuantStudio™ Real-Time PCR Software v1.3 and exported to an XLS file for further analysis. Threshold was set to the same value for all targets across the experimental runs.

**Table 2:** Primers and probes used for qPCR assay with corresponding annealing/extension temperatures and amplified fragment length.

Organism	Target	Oligo ID	Sequence 5’-3’	Length (nt)	Fluorophore	Quencher	Ta (°C)	Fragment (nt)
Arsenophonus	16S rRNA	PRA-Ars1-F	TATCGCAGGAGAAAAGTCTG	20	–	–	60	91
Arsenophonus		PRA-Ars1-R	GCCCTACTCTTTGAGTTCAC	20	–	–		
Arsenophonus		PRA-Ars1-P	ACCGGCAATAAAGGGTAATAGCCC	24	TAMRA	BHQ®-2		
Portiera	16S rRNA	PR-Port1-F	GCAGAAGAGGAAGGTAGAA	19	–	–	60	110
Portiera		PR-Port1-R	TTCGCATCTCAGTGTCAG	18	–	–		
Portiera		PR-Port1-P	CGCCTTCGCAACTGGTATTCC	21	HEX	BHQ®-1		
Rickettsia	gltA	PR-Rick1-F	CTCCGCAAATGTTCACAG	18	–	–	60	102
Rickettsia		PR-Rick1-R	GTCTGCTGATTTTCTGCTC	19	–	–		
Rickettsia		PR-Rick1-P	TCCATTGTGCCATCCAGCCTA	21	6-FAM	BHQ®-1		
*B. tabaci*	Actin	SX-Actin1-F	CCCATCTACGAAGGTTAC	18	–	–	55	98
*B. tabaci*		SX-Actin1-R	CGTTCAGTGAGGATTTTC	18	–	–		
*B. tabaci*		SX-Actin1-P	TCAAGTCACGACCAGCCAAGT	21	6-FAM	BHQ®-1		
*B. tabaci*	COX1	SX-COX1-F	GGTGGTTTTGGTAATTGG	18	–	–	55	98
*B. tabaci*		SX-COX1-R	GAAGGAACTAAAAGTCAAAAC	21	–	–		
*B. tabaci*		SX-COX1-P	CCTCTGATAATTGGTGCTCCTGACA	25	HEX	BHQ®-1		

### Quantification of rifampicin

To test the validity of rifampicin delivery through the plant and into the insect body, a separate assay was performed to detect and quantify the antibiotic in both plant and insect tissue. Rooted tomato side shoots were immersed in opaque 250 ml glass bottles containing a 25 mg/l rifampicin water solution. About 150 whitefly adults were introduced 72 h later in a clip cage clipped to a single tomato leaflet. At this point first tomato leaf samples were collected, weighed, flash frozen in liquid nitrogen and stored at −80 °C until processing. 96 h later, whitefly adults were removed, counted, flash-frozen in liquid nitrogen and stored at −80 °C. Tomato leaflets to which whiteflies were restricted were also collected, weighed, and stored in the same way. Separately, 230 whitefly individuals were weighed to determine the average weight of unsexed whitefly adult.

Rifampicin was extracted from the leaf and whitefly samples using LC–MS grade acetonitrile (ACN) as follows. Samples were frozen in liquid nitrogen and ground in 2 ml tubes using bead mill homogenizer with two 3 mm stainless steel balls for 1 min at the frequency of 30 Hz, with liquid nitrogen pre-cooled tube holders. Samples were then briefly centrifuged, acetonitrile was added to the tubes, and the entire sample was transferred to a glass test tube. Steel balls were removed using a magnet and washed with acetonitrile which was also added to the sample. Samples were then placed in an ultrasonic bath Elmasonic S300 (Elma Schmidbauer GmbH, Singen, Germany) for 15 min. Liquid was aspirated and filtered through a 0.45 µm PVDF syringe filter into a new glass vial. Pellet of solid parts from the tomato leaf samples was subjected to a second round of extraction by adding 2 ml of acetonitrile and sonicating for 15 min, followed by aspiration of the supernatant and filtering, and was added to the extract from the first round. Samples (about 5 ml total per sample) were then evaporated at 30 °C with 1–2 l/min airflow until dry using TurboVap® LV evaporator (Biotage Sweden AB, Uppsala, Sweeden) and reconstituted in 2 ml of 1:10 ACN: H_2_O. Samples were diluted 1:200 and 1:40,000 in 1:10 ACN: H_2_O and placed in the autosampler of the LC–ESI–MS/MS system Agilent HPLC (Agilent, Santa Clara, USA) coupled with a QTRAP 4500 MS/MS (AB Sciex, Framingham, USA) for quantification of rifampicin. An online-SPE injection method (0.9 mL injection volume per sample) was used. Online extraction of the water samples was performed using trapping column Hypersil Gold, 20 × 2.1 mm and 12  m particle size (C18 Selectivity phase, Thermo Fisher Scientific). Separation of rifampicin was performed using Luna Omega C18 Polar column (100 × 2.1 mm with 3  m pore size) (Phenomenex) in positive ionization mode. A mass spectrometry grade rifampicin was used for preparing the standards. A seven-point calibration was prepared and measured before measuring the samples. After ten samples a quality control sample and a blank were injected to control the system stability. The achieved quantification range was 10–1000 ng/l.

### qPCR data analysis

A custom R script, loosely based on the R “pcr” package of Ahmed and Kim^42^, was developed to create standard curves and determine relative quantity of each target based on the Ct values exported from the QuantStudio software. Linear model was fitted on the 10-point serial dilution Ct values and log10 transformed serial dilution quantities. Slope, intercept, R squared, and p-value were calculated, and standard curves plotted. Intercept and slope of the curve were then used to calculate relative quantities from the Ct values of each sample and each target. In the case when quantity normalization by another target is applied, the quantity of the target to be normalized was divided by the quantity of the reference target for that sample. Then, to meet the normality assumption of analysis of variance (ANOVA), box-cox power transformation was applied to the relative quantities using optimal lambda parameter. Finally, ANOVA with post-hoc Tukey test was performed to compare the differences between treatment groups and results graphed using box and whiskers plot and exported to the CSV file. Additional box and whiskers plot of raw Ct values was generated. In case of Arsenophonus, there were specimens in all treatment groups which tested negative for Arsenophonus. The numbers of negative samples per group were statistically compared using pair-wise Fishers exact test on a two-way contingency table as implemented in “rcompanion” R package. All R scripts described and used in the present study are available in the supplementary material as well as at the public GitHub repository (https://github.com/milenovic/rifampicin-qPCR-analysis-R). Finally, the ratio of relative quantities between males and females was calculated in Microsoft Office Excel by dividing the mean relative quantity of males by the mean relative quantity of females for each target and treatment group.

## Results

### Whitefly holobiont identity

PCR amplification of whitefly mtCOI gene fragment followed by sequencing produced identical sequences across 10 sampled individuals and yielded the sequence of 1175 bp. BLAST search against NCBI Nucleotide database showed 100% identity with MH205753.1 which is labelled as *Bemisia tabaci* MED Q2 mitochondrion. Subsequent phylogenetic analysis grouped our population closely with *B. tabaci* MED found in Cyprus, Israel, and Syria (Figure S3 of the supplementary material). Sequence obtained using Portiera specific primers (811 bp after trimming) showed 100% identity with several accessions, including the accession AB981341 which is Portiera group P1 according to Kanakala and Ghanim^11^. Sequence obtained using Rickettsia specific primers produced a trimmed sequence of 859 bp having 100% identity with accession KM386372, which is whitefly endosymbiont Rickettsia R1 group according to Kanakala and Ghanim^11^. Sequence obtained using Arsenophonus specific primers yielded a trimmed sequence of 425 bp. The top BLAST hit of this sequence was the sequence under the accession number FJ766366.1 with 97.88% identity. This accession is identified as *B. tabaci* Arsenophonus endosymbiont isolated from the population from Burkina Faso. As identical hit was not found, a phylogenetic analysis was performed. As shown in the Figure S4 of the supplementary material, Arsenophonus from this study groups closest to accession FJ766366 which is Arsenophonus group A2c according to Kanakala and Ghanim^11^. Despite being closest to the group A2c, phylogenetic analysis indicates that this Arsenophonus strain belongs to a distinct, previously undescribed subgroup. The produced Arsenophonus sequence is deposited to the GenBank database under the accession number OP289131.

**Figure 1:**
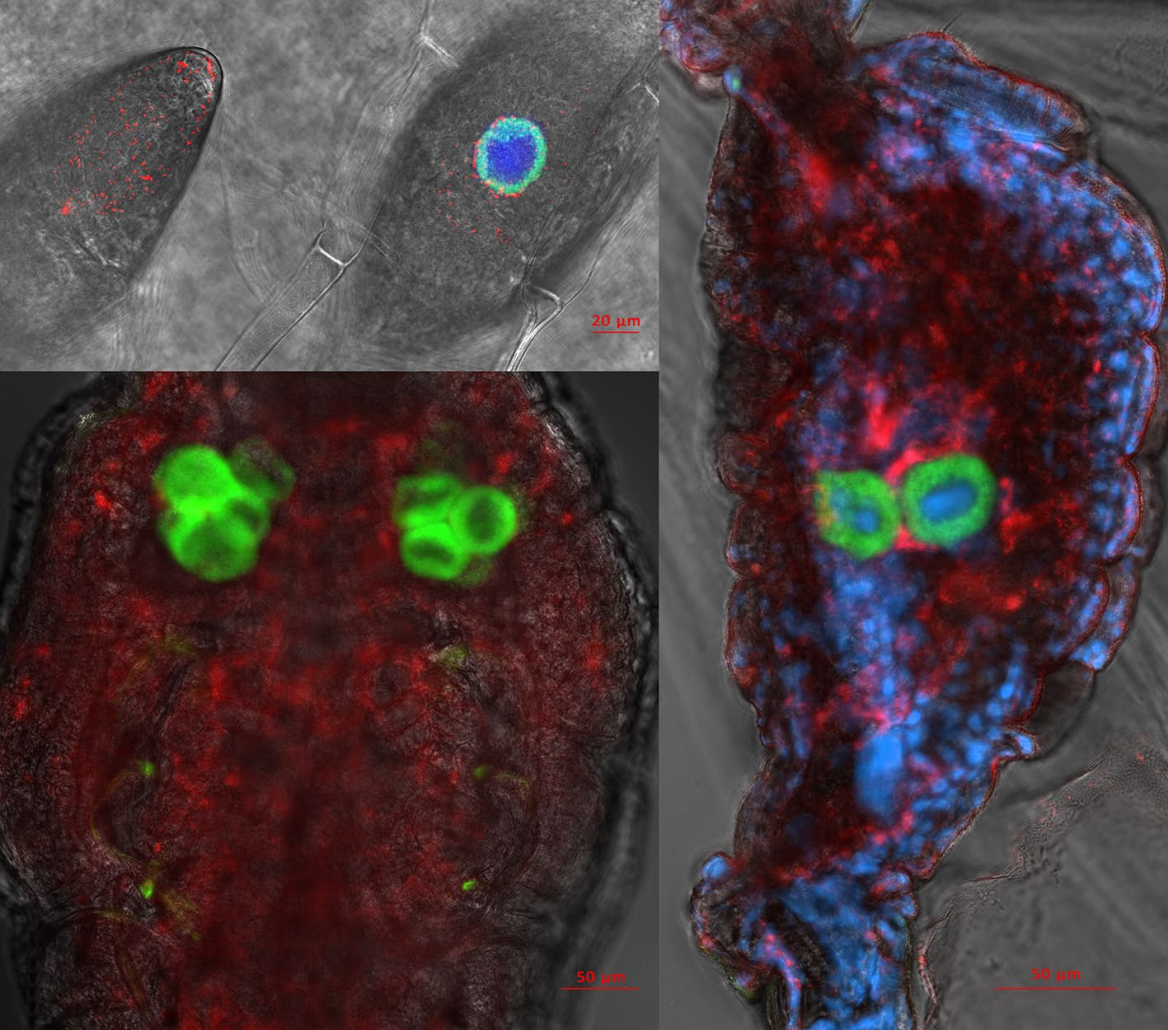
Localization of endosymbionts Portiera (green) and Rickettsia (red) in whitefly eggs (top left), nymphs (bottom left), and adult abdomen (right) using fluorescence in situ hybridization with DAPI nucleic acid counterstain (blue). DAPI was not used in the nymph sample. The background grayscale image shows the transmitted light acquired using the transmitted photomultiplier tube (tPMT) detector.

**Figure 2:**
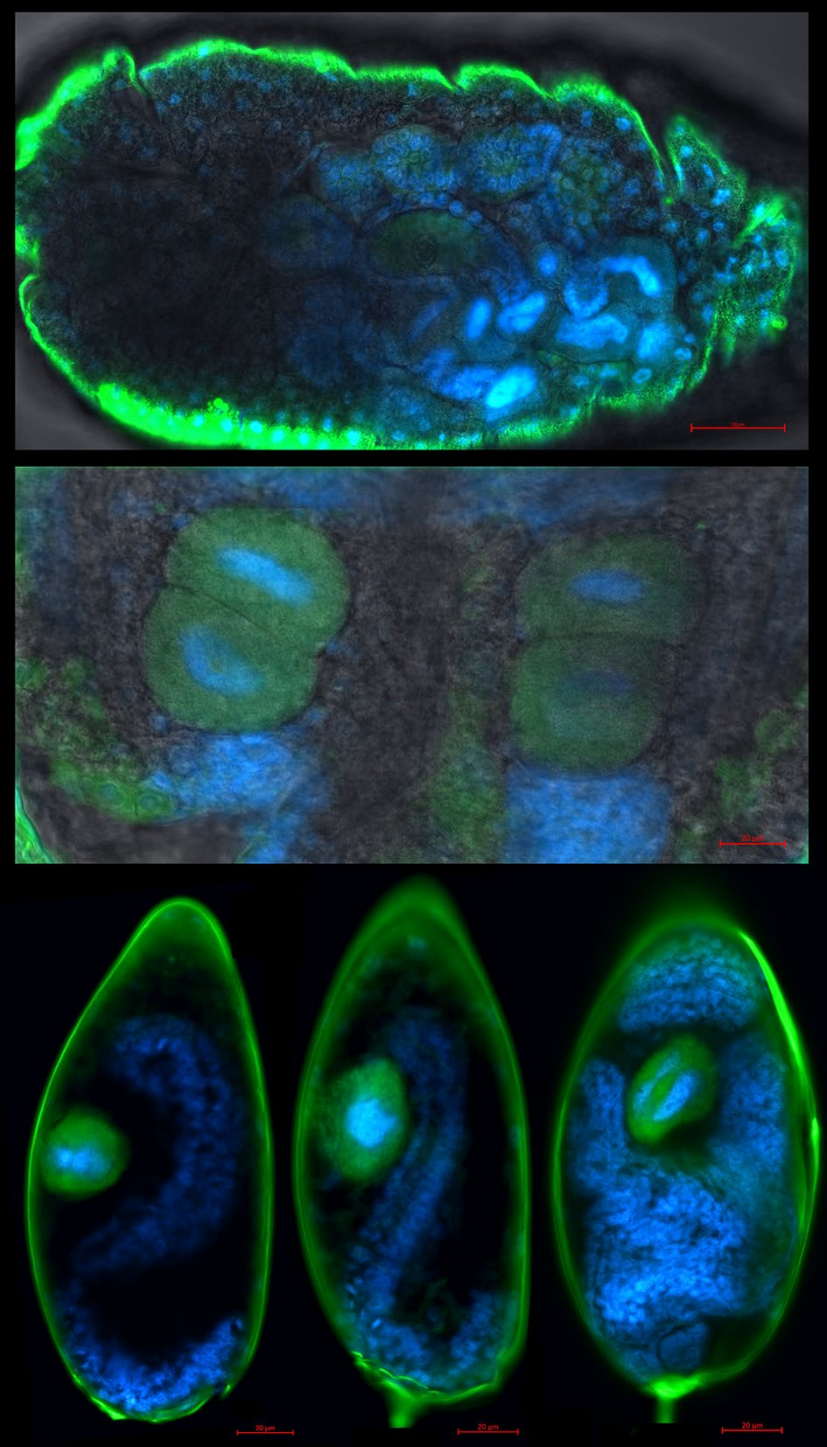
Localization of Arsenophonus (green) in whitefly adults (top), nymphs (middle), and eggs (bottom three) using fluorescence in situ hybridization with DAPI nucleic acid counterstain (blue). Note the strong false signal around the edges of the samples caused by the refraction of laser light when passing through the cuticle. This artefact is especially pronounced here due to high laser power and gain that were required to detect low densities of Arsenphonus. The background grayscale image shows the transmitted light acquired using the transmitted photomultiplier tube (tPMT) detector.

Localization of endosymbionts reveals typical bacteriocyte-confined phenotype for Portiera, while Rickettsia is localized primarily in haemolymph, but is also clearly present in bacteriocytes (Fig. 1). This was the case for all developmental stages, from the egg to adult. Novel Arsenophonus strain was localized to bacteriocytes (Fig. 2). Localization of Arsenophonus points towards very low densities of this endosymbiont in whiteflies, as the signal was barely detectable even with high laser power, high signal gain, and despite using doubly labelled probe which additionally increases the signal intensity.

### Endosymbiont densities and effects of normalization

Standard curves used for relative quantification are presented in the supplementary material. PCR resulted in amplification of all targets in all ten dilutions, except for Arsenophonus, where the last dilution resulted in no amplification. Raw Ct values (Figure S2 in the supplementary material) give an overview of the quantities between the treatment groups and provide a rough indication of quantities between different targets. For all targets, a general pattern of lower Ct value can be observed in females than in males.

**Figure 3:**
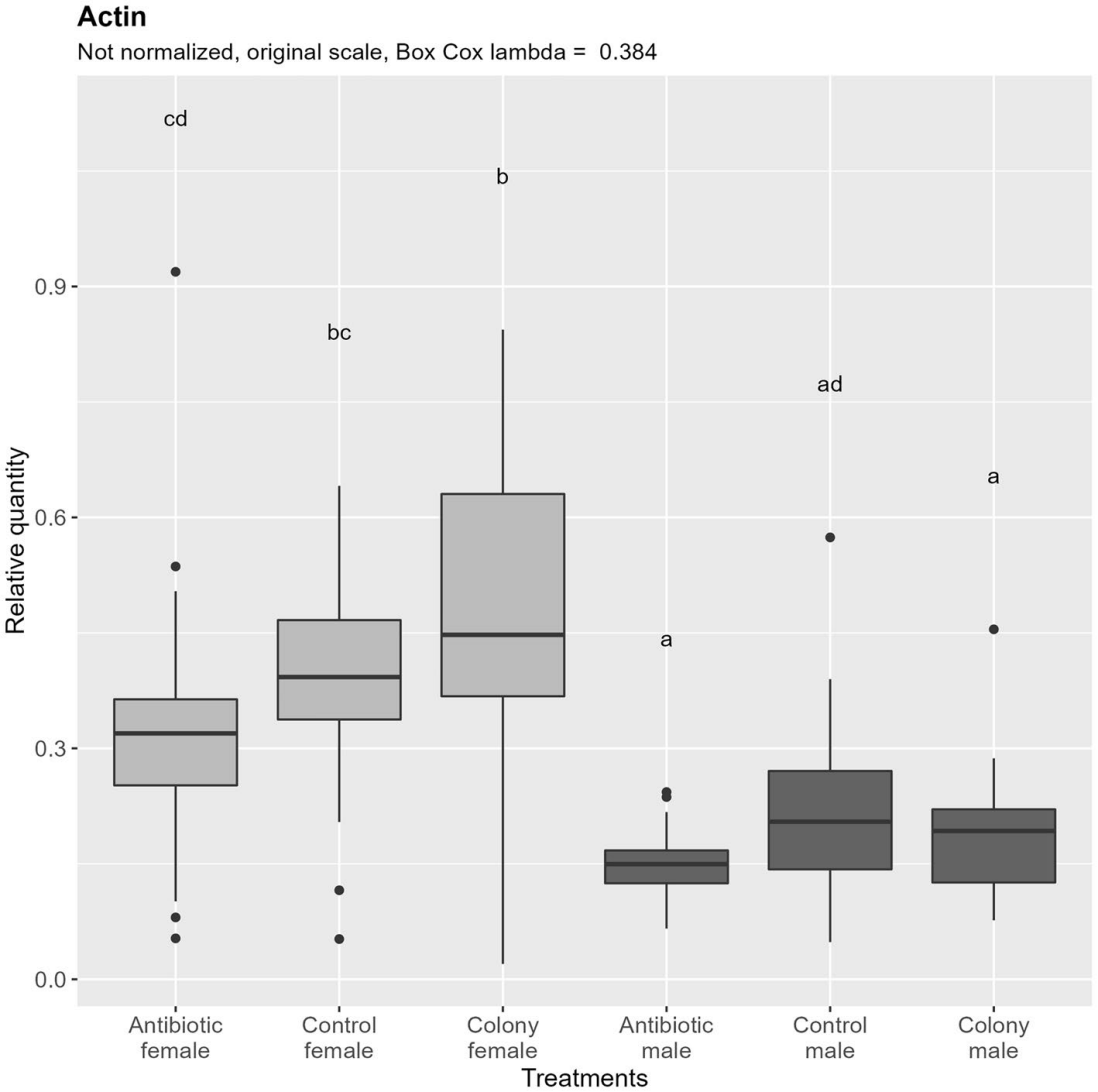
Relative quantification of nuclear DNA (Actin target) across the treatment groups. Note that the relative quantity is shown on the original scale for more intuitive interpretation, while the ANOVA was performed on the Box Cox transformed data. Values with the same letter are not significantly different (alpha = 0.05, Tukey’s HSD test). Boxes represent the data between 25 and 75th percentile, horizontal line within the box represents median, and dots represent the outliers. A data point was considered an outlier as per the standard definition of the R package ggplot2 (when the data point (x) is either lower than Q1—1.5 * IQR (interquartile range) or greater than Q3 + 1.5 * IQR). Vertical lines extending the boxes (whiskers) are drawn using standard ggplot2 function and are calculated using the following formulas: upper whisker = min(max(x), Q3 + 1.5 * IQR), lower whisker = max(min(x), Q1—1.5 * IQR). IQR = Q3—Q1. Quantiles are calculated according to the default type 7 definition in R.

Quantification of nDNA (Fig. 3) shows significant differences between males and females, which nearly perfectly correspond to the theoretical double the amount of nDNA in females due to their diploid nature. Comparing between the treatment groups, no significant differences are observed between antibiotic treated and control groups in both males and females. No significant difference is observed between colony and control groups in both sexes. A difference can be observed in females, where nDNA content in the antibiotic treated group is significantly lower than in the colony, but not compared to the control. This difference could be explained by the different and uncontrolled age of the collected colony adults.

**Figure 4:**
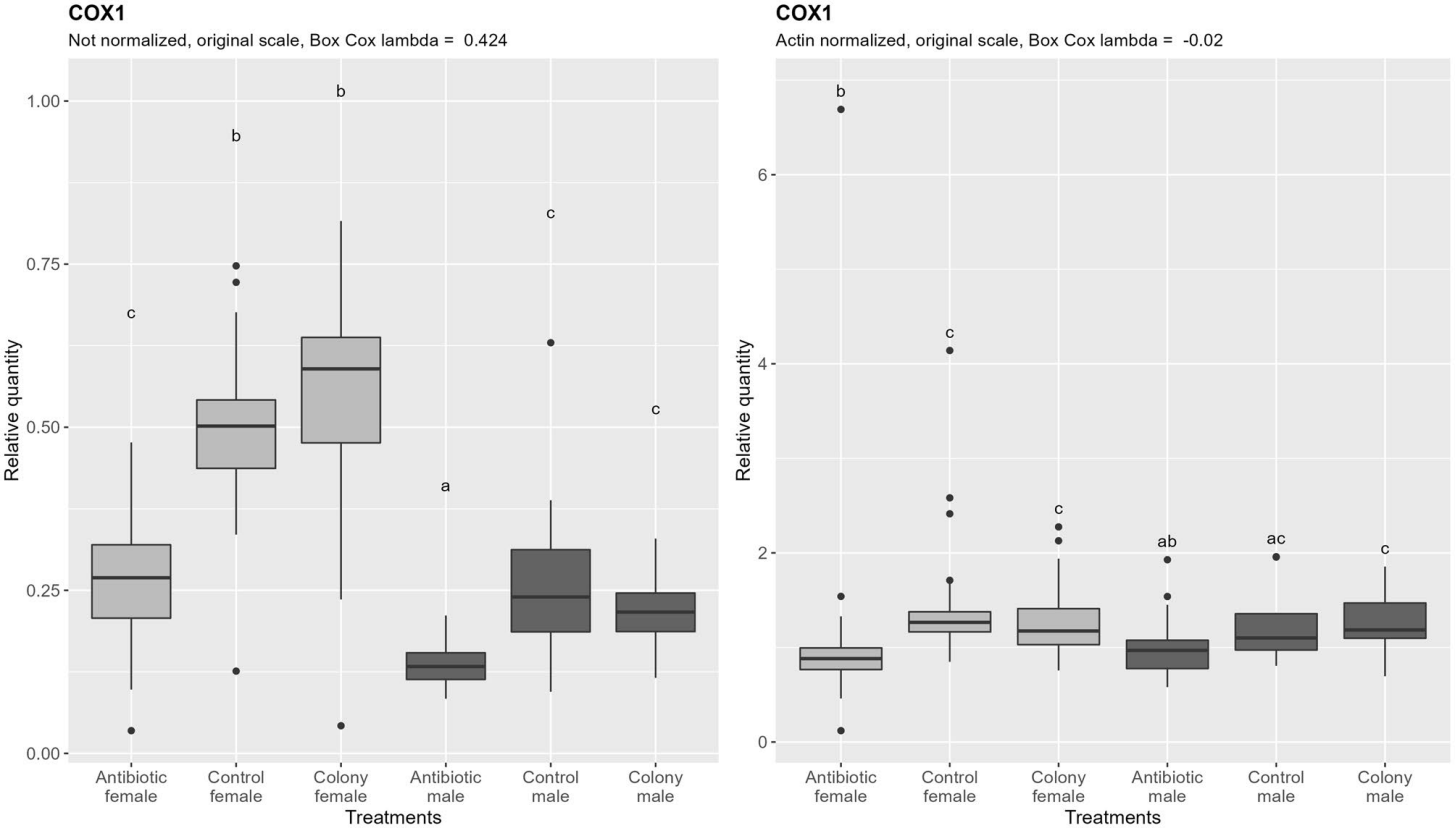
Relative quantification of *B. tabaci* mitochondrial DNA (COX1 target) across the treatment groups. Figure on the right represents quantities after normalization to the nuclear DNA, while the figure on the left shows the data without normalization. In both cases, ANOVA was performed on the Box Cox transformed data. Within one figure, values with the same letter are not significantly different (alpha = 0.05, Tukey’s HSD test). Note that the relative quantity (y-axis) is shown on the original, not transformed scale for more intuitive interpretation. Definition of the boxplot is the same as in the Fig. 3.

Quantification of mtDNA shows no difference between control and colony for both sexes, as in the case of nDNA, and similarly, about 50% less mtDNA is present in males. Antibiotic treatment affected the amount of mtDNA, as seen by a significant reduction of mtDNA in antibiotic treated groups, contrary to the case of nDNA (Fig. 4, left). If the ploidy influenced effect on mtDNA content is subtracted by normalizing the results by nDNA (Fig. 4, right), overall variability in the data is reduced, and the mtDNA content difference between control/colony and antibiotic treated groups remains in females, while it is not significant in males, although it does show a trend.

**Figure 5:**
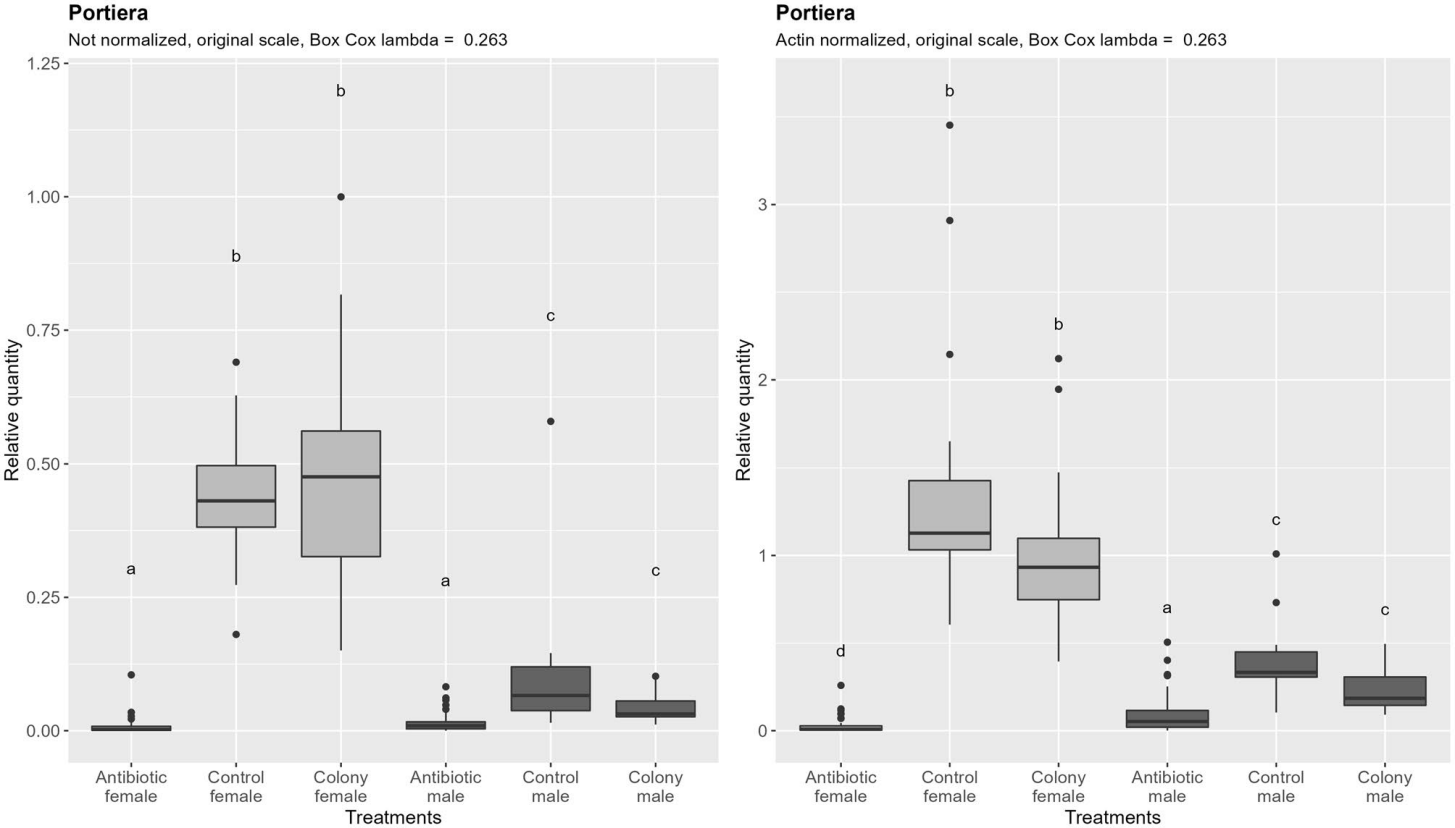
Relative quantification of Portiera across the treatment groups. Figure on the right represents quantities after normalization to the nuclear DNA, while the figure on the left shows the data without normalization. In both cases, ANOVA was performed on the Box Cox transformed data. Within one figure, values with the same letter are not significantly different (alpha = 0.05, Tukey’s HSD test). Note that the relative quantity (y-axis) is shown on the original, not transformed scale for more intuitive interpretation. Definition of the boxplot is the same as in the Fig. 3.

Quantification of Portiera densities reveals more striking significant difference between males and females (Fig. 5, left), which still remains after the subtraction of the ploidy effect, by normalizing to the quantity of nDNA (Fig. 5, right) on a per-sample basis. Antibiotic treatment caused a 129-fold reduction of Portiera in females and sixfold reduction in males when compared to the control (normalized data). After antibiotic treatment, there was no significant difference between males and females in not normalized data although a trend of lower densities in females is visible. After normalization, however, the significantly lower densities of Portiera in females compared to males are apparent. Taken together, there was a difference in the efficacy of rifampicin in reducing densities of Portiera between females and males. The densities of Portiera do not correspond exactly to the difference if nDNA content as it is the case for mtDNA.

**Figure 6:**
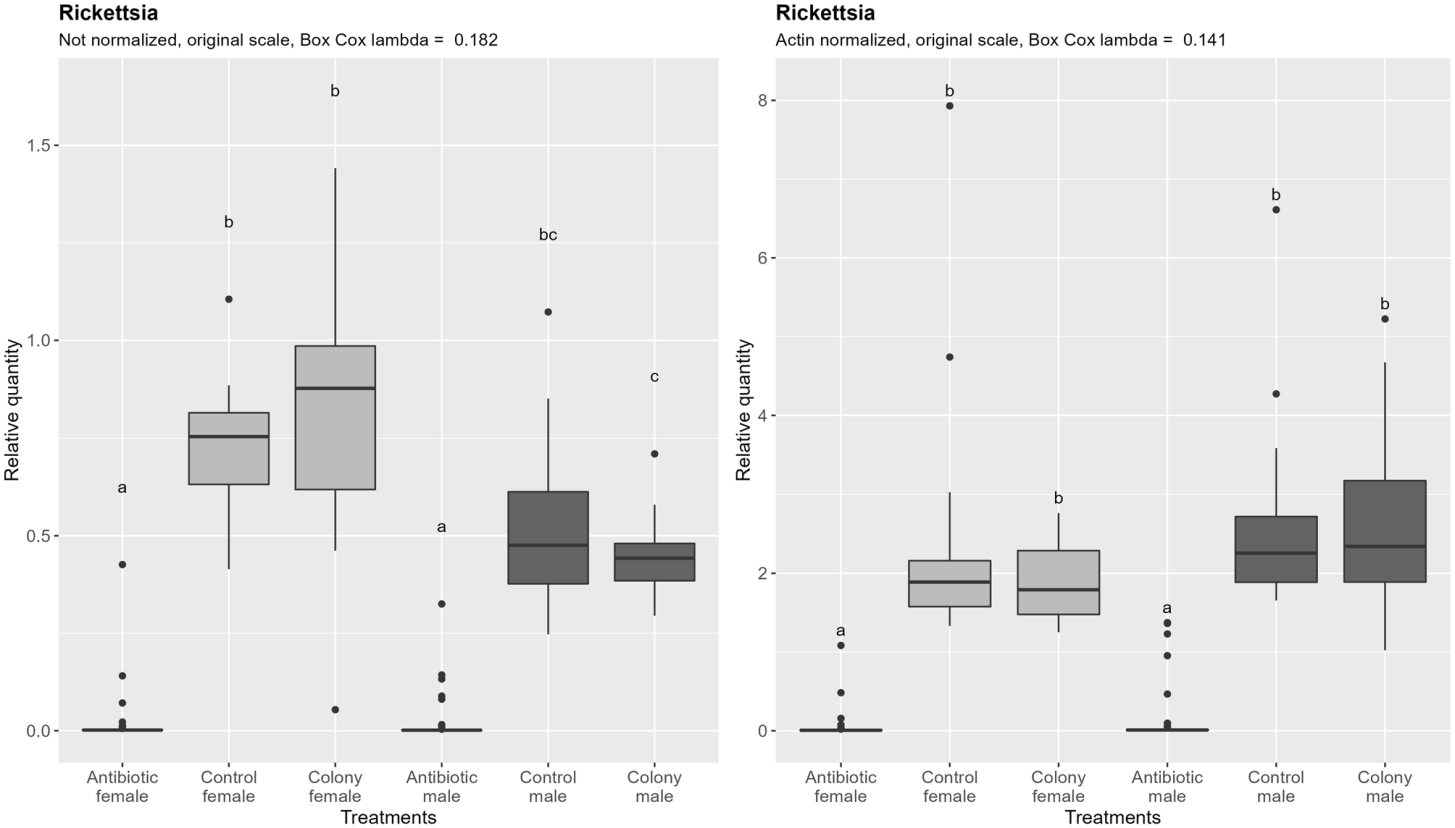
Relative quantification of Rickettsia across the treatment groups. Figure on the right represents quantities after normalization to the nuclear DNA, while the figure on the left shows the data without normalization. In both cases, ANOVA was performed on the Box Cox transformed data. Within one figure, values with the same letter are not significantly different (alpha = 0.05, Tukey’s HSD test). Note that the relative quantity (y-axis) is shown on the original, not transformed scale for more intuitive interpretation. Definition of the boxplot is the same as in the Fig. 3.

Contrary to Portiera, Rickettsia does not show significant different densities between males and females, although a non-significant trend to lower densities in males exists before the data are normalized by the nDNA content  (Fig. 6). After accounting for the ploidy effect by normalization, the still non-significant trend is in opposite direction. Antibiotic treatment resulted in 297-fold reduction in females and 223-fold reduction in males compared to the control (normalized data). There was no significant difference between males and females after antibiotic treatment regardless of the normalization.

**Figure 7:**
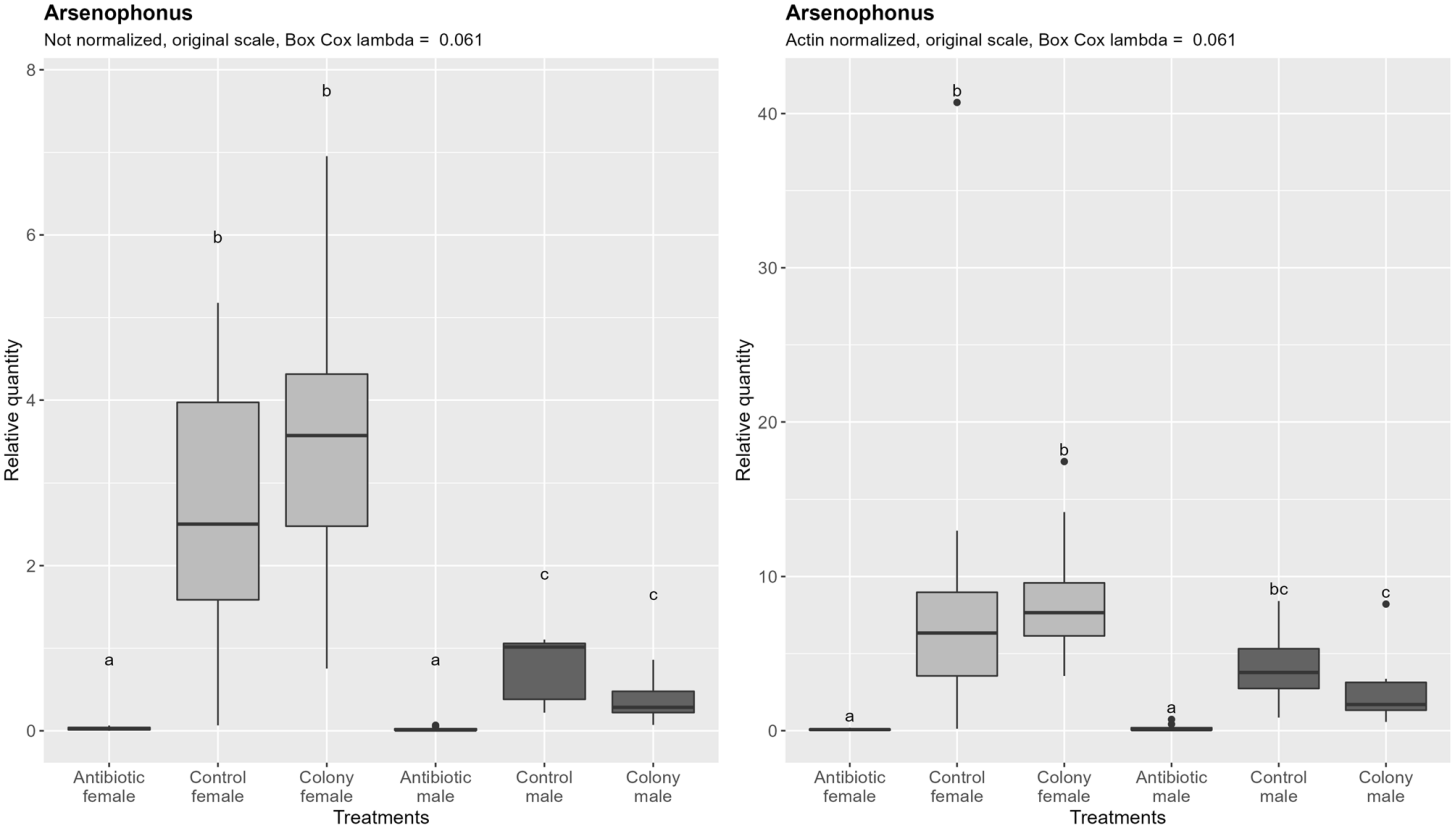
Relative quantification of Arsenophonus across the treatment groups. Figure on the right represents quantities after normalization to the nuclear DNA, while the figure on the left shows the data without normalization. In both cases, ANOVA was performed on the Box Cox transformed data. Within one figure, values with the same letter are not significantly different (alpha = 0.05, Tukey’s HSD test). Note that the relative quantity (y-axis) is shown on the original, not transformed scale for more intuitive interpretation. Definition of the boxplot is the same as in the Fig. 3.

Arsenophonus densities are significantly higher in females in both colony and control groups  (Fig. 7). The difference becomes less pronounced after subtracting the effect of ploidy level, and in the case of the control and antibiotic treated group it becomes statistically non-significant. Antibiotic treatment resulted in 87-fold reduction in females and 56-fold reduction in males compared to the control (normalized data). There was no significant difference between males and females after antibiotic treatment regardless of the normalization. Comparison of the proportion of Arsenophonus negative to positive reactions between treatments showed no statistically significant difference (Tables S1 and S2 in the supplementary material).

Microscopy observations of antibiotic treated individuals revealed much weaker signal intensity of Portiera, barely detectable signal of Rickettsia specific probe, and undetectable signal from Arsenophonus specific probe. Antibiotic treated nymphs and adults appeared paler in color (less pronounced yellow color) when observed under visible light stereo microscope. In antibiotic treated nymphs, there seemed to be more individuals with asymmetric or only one bacteriocyte present (data not quantified).

An attempt was made to produce the second generation from the antibiotic treated whiteflies on fresh, untreated plants. However, the efforts failed as only very few eggs were oviposited and these failed to develop.

### Rifampicin detection

Presence of rifampicin was detected in both plant leaves before whitefly introduction and after whitefly sampling. Antibiotic was also detected in whitefly samples although in much lower amounts (about 7000-fold lower) when comparing antibiotic mass per mass of the sample. We measured the mean weight of an unsexed whitefly individual to be 37.4 ng. We were unable to obtain absolute quantity of rifampicin with confidence due to the dual rifampicin peaks in liquid chromatography separation in the antibiotic treated samples. The same double peak appeared as well as in the standards, but with a different ratio between the peaks. Rifampicin concentration in whiteflies seemed to be correlated to the concentration of antibiotic in the leaves that they were feeding on (r = 0.801, n = 7) although a higher number of samples would be needed to statistically evaluate this relationship.

## Discussion and conclusion

### Characterization of whitefly holobiont is important

Complete characterization of whitefly holobiont, which includes phylogenetic placement of the whitefly, composition, and phylogeny of its obligatory and facultative endosymbionts, as well as their localization, can seem as a time-consuming process without immediate benefits in studies focused on only one aspect, such as whitefly life table. However, adoption of such characterization greatly improves data comparability across different studies and holds the potential to over time reveal large scale patterns that would otherwise remain a mystery. Characterization of the population used in the present study, collected in Sicily (Italy), identifies the holobiont as *Bemisia tabaci* MED, with its obligatory endosymbiont from the P1 group and two facultative endosymbionts, haemolymph localized Rickettsia from the R1 group, fixed in the population, and a novel strain of bacteriocyte-confined Arsenophonus from a group similar to the A2c group, present in 50–70% of individuals. The discovered Arsenophonus strain expands the known diversity of whitefly endosymbionts and suggests how already complex evolutionary history of Arsenophonus might not be fully described^11,43^. The sequences of whitefly endosymbionts presented here are the first published from Italian whitefly populations. The detection of a novel strain in the sample of just one population sparks the question on how much diversity is yet to be uncovered in poorly sampled regions of whitefly geographic distribution.

### Rifampicin severely reduces but does not eliminate Portiera, Rickettsia and Arsenophonus

In the present study, egg-adult treatment with a high concentration of rifampicin failed to eliminate any of the three present endosymbiont species even from a single individual, which is in agreement with the study of Shan, et al.^18^. The substantial reduction of all endosymbionts was observed and ranged between 6 and 297-fold. Different species show different magnitudes of reduction. Rickettsia had the highest observed reduction (250-fold) similar in both sexes, followed by Portiera in females (87-fold), Arsenophonus in both sexes (around 70-fold), and curiously, only sixfold reduction of Portiera in males. The magnitude of the reduction appears to be linked with the initial densities of the endosymbionts. Arsenophonus was definitively present in very low quantities as seen both by semi-quantitative microscopy observations of FISH samples and by much higher Ct values for Arsenophonus in qPCR assays compared to the other targets. Similarly, a discrepancy in reduction of Portiera in females and males is partly explained by 3.4 times higher density in control females compared to control males. This link between initial quantities and fold change is however not perfect. Further, higher reduction of more abundant bacteria is counterintuitive at first as one would expect either no link, or the opposite link due to the so called “inoculum effect”, where the antibiotic efficacy can be reduced in higher bacterial densities^44^. Finally, our results show the benefit of accurate quantification of endosymbiont densities as opposed to expressing “percent of infected individuals” which is often used instead in the antibiotic experiment studies. Quantification not only provides much deeper insights in antibiotic effects but also allows better comparisons of results between studies. Similarly, we stress the importance of testing evaluating antibiotic effects on males and females separately. Lack of quantification data, sex-specific data, and use of parafilm feeder antibiotic delivery method, renders the previous studies less detailed in comparison to the present work and makes it hard to draw meaningful comparisons.

### Whitefly endosymbionts differ in their sensitivity to rifampicin

We hypothesize that the differences in reduction between endosymbiont species come mainly from the different intrinsic sensitivity to rifampicin between the species, and to much lesser extent from the different localization. Portiera and Arsenophonus are both bacteriocyte confined yet show different response to rifampicin. This supports the findings of Zhao, et al.^15^ who showed that localization influences the efficacy of ampicillin, but not rifampicin which is able to more efficiently cross cell membranes. High sensitivity of Rickettsia towards rifampicin is unsurprising, considering that rifampicin is the antibiotic with one of the lowest minimum inhibitory concentration across 27 tested Rickettsiae^45^. Despite their different sensitivity we were unable to verify the findings of Zhao, et al.^15^ that Portiera is reduced at a slower and different enough rate to allow selective elimination of secondary endosymbionts. The present study used four times less concentrated rifampicin solution and failed to produce viable offspring from egg-adult antibiotic treated individuals, which is again the same outcome as the one of Shan, et al.^18^ and Zhang, et al.^19^. Perhaps shorter but repeated exposures to rifampicin over the course of several generations would yield a different result. For now, we conclude that selective and stable elimination of Rickettsia and Arsenophonus remains a difficult task. The antibiotic treatments of whiteflies in previous studies yielded mixed results, and different strains of the same endosymbiont might have differential response to the same treatment.

### Host sex influences effect of rifampicin on Portiera

It appears that sex-specific reduction of Portiera could be explained by the reduction of the number of bacteriocyte cells, more specifically, by the lower number of eggs present in antibiotic treated females, each of which inherits one bacteriocyte cell during the egg development. The significant differences in Portiera densities between females and males exist even after subtracting the effect of the ploidy level by normalizing by the nDNA content, which hits towards this conclusion. However, egg end bacteriocyte counts were out of the scope of the present study. Even if the hypothesis is true, and absolutely no eggs were present in antibiotic treated females, the post-antibiotic males and females would be expected to have similar quantity of Portiera. The present study shows that this is not the case. Antibiotic treated females have sixfold lower Portiera density compared to antibiotic treated males (Fig. 5). A further study focused on nymphs could be performed to further test the role of eggs in sex-dependent reduction of Portiera following the rifampicin treatment. One would expect no significant differences between sexes in rifampicin-induced reduction of Portiera. Finally, the sex-specific reduction of Portiera by rifampicin treatment is novel in the scientific literature as there are currently no studies that quantified endosymbiont densities separately for the two sexes and used plant feeding method of antibiotic delivery that allows antibiotic exposure longer than a few days.

### Normalizing the data reduces variability and offers insights in cellular metabolic scaling

The qPCR normalization method, which is a standard practice in gene expression (qRT-PCR) studies. In quantifying non-nuclear DNA however, normalization is often not performed. The present study improves the method by normalizing mitochondrial and bacterial DNA quantifications with nuclear DNA as a reference. We demonstrate how such normalization in individual whiteflies reduces the overall data variability. The method assumes that nDNA content between whitefly individuals of the same sex varies less than the efficiency of DNA extraction from a single whitefly using manual grinding methods. Indeed, when comparing within same sex individuals, the data is less dispersed. Further, we take a step further and show how normalization by nDNA in haplodiplod species can be used to probe the relationship between ploidy level and non-nuclear DNA contents, which in the present study reveals an intriguing link between nDNA content, endosymbiont density, and localization. A caution however must be exercised when comparing normalized values between individuals with different ploidy level. Such comparisons must be limited to studying the sex-specific influence of the treatment, and to studying the relationship between ploidy level and non-nuclear DNA target quantity.

The ratio of nDNA between haploid males and diploid females is within the margin of error from the theoretical 1:2. The same ratio is however observed for the mtDNA content, which is in perfect alignment with previous observations of conserved linear increase of mtDNA with the increase in ploidy in yeast (*Saccharomyces cerevisiae* Meyen ex E.C. Hansen) and grass carp (*Ctenopharyngodon idella* Val.)^46,47^. Similar conserved ratio seems to also exist between nDNA and chloroplast DNA in the unicellular green alga *Chlamydomonas reinhardtii* P.A. Dang^48^. As shown in Fig. 7 and Table 3, density of Rickettsia is also approximately double in diploid females, and when quantities are normalized by nDNA, differences among sexes become non-significant. The fact that Rickettsia density follows the same rules of cellular metabolic scaling as mitochondria and chloroplasts points towards shared underlying factors that dictate their optimal cellular densities.

**Table 3:** Male: female ratio of qPCR targets quantities across three treatments.

	nDNA	mtDNA	Arsenophonus	Portiera	Rickettsia
Colony	0.40	0.40	0.11	0.09	0.54
Control	0.59	0.52	0.29	0.23	0.71
Antibiotic	0.47	0.51	0.79	1.72	1.13

Bacteriocytes, being inherited from the mother in both sexes without expected changes in their ploidy, should contain equal amount of nDNA in both sexes. However, density of bacteriocyte confined endosymbionts Portiera and Arsenophonus still differ between sexes and follow a very different nDNA ratio. This is evident from the fact that differences between sexes largely remain after accounting for the differences in nDNA content by normalization, and by numeric representation in the Table 3. Males have less than a third of the density of both Portiera and Arsenophonus compared to females. Data presented in this study is insufficient to fully understand why the difference exist and why it takes the value that it does. We hypothesize that the difference comes from the larger total volume of bacteriocytes in females. A follow-up study examining the ratio of their density and nDNA content in whitefly nymphs, including determining bacteriocyte sizes and counts, could provide the means of testing our hypothesis.

### Delivery of the antibiotic is not a limiting factor of its efficacy

Detection of rifampicin in plant leaves and whitefly bodies confirms that (a) observed reduction of endosymbiont densities is a direct effect of the antibiotic, (b) long-term delivery through the plant is a superior method compared to the delivery through the artificial feeder due to the prolonged time of exposure, which is not possible on artificial diet. We hypothesize that the dual peaks of rifampicin in our liquid chromatography separation result from alternative forms of rifampicin, most likely rifampicin quinone as previously reported in the literature^49^. While absolute quantification would have been nice addition to the present study, we decided not to pursue it as the detection was sufficient to confirm that the antibiotic was indeed taken up by the plant cuttings and ingested by the whiteflies.

### Rifampicin negatively affects whitefly mitochondria

Mitochondria, famously described as the powerhouse of the cell^50^, are as critical to the metabolism of almost all eukaryotes as they sound and their disruption will affect many aspects of the metabolism. By quantifying both nuclear and mitochondrial DNA control and antibiotic treated samples, we were able to demonstrate no significant differences in nuclear DNA content between treated and control individuals, and significant differences in mitochondrial DNA (Fig. 4, left). Further, after accounting for the ploidy related differences by normalizing mtDNA by the nDNA, sex-specific differences become non-significant, while the significant difference remains between antibiotic treated and untreated females (Fig. 4, right). In case of males after normalization, the difference is not significant, although a trend exists. This clearly shows that reduction in mtDNA content in females is due to the rifampicin treatment. These results indicate that rifampicin might have a direct negative effect on mitochondria and poses a question of its use in temporary reduction of whitefly endosymbionts followed by the measurements of whitefly biology parameters, especially if the observed effects are then interpreted as consequences of manipulated endosymbiont community. Alternatively, it is possible that reduction in mtDNA content is an indirect response to the reduced endosymbiont densities, and not direct effect of rifampicin. Further studies with dedicated methodology for studying mitochondrial disruption are needed to determine the underlying mechanism. Nevertheless, interpretations of the previous studies employing such methodology must be approached with caution. Rifampicin could influence the biology of the whiteflies more than the presence of secondary endosymbionts.

### Antibiotics and the future of endosymbiont manipulation methods

Antibiotics have been an important tool in the early endosymbiont research and leave a legacy which all modern research is built upon. Antibiotic experiments, especially rifampicin, very early on demonstrated the crucial role of the primary endosymbiont and shown how its disruption is a valid and promising approach to whitefly control. Now, a method for disrupting endosymbionts without using antibiotics is needed in order to exploit the knowledge for developing whitefly control methods. With the potential of whitefly antibiotic treatment as a research tool mostly realized, it is time to move on to the use of new, more targeted antibacterial compounds in whitefly endosymbiont research. Antibiotic treatments have shown us that even severely reducing endosymbiont densities severely reduces the fitness of whiteflies, similarly to the previous attempts to create quasi-aposymbiotic *Blattella germanica* L.^51^. With the recent advances in the field of antimicrobial peptides and gene silencing technologies, the future looks bright for the novel whitefly control methods.

## Supplementary Information

Supplementary Information 1.

Supplementary Information 2.

Supplementary Information 3.

Supplementary Information 4.

## Data Availability

Sequence generated in this study is available at the NCBI GenBank database, https://www.ncbi.nlm.nih.gov/nuccore/OP289131. All other data generated or analysed during this study are included in this published article (and its Supplementary Information files).
