# Supplementary material for: Plant-mediated rifampicin treatment of Bemisia tabaci disrupts but does not eliminate endosymbionts
Source: Sci Rep. 2022 Dec 1;12:20766. doi: 10.1038/s41598-022-24788-0 (PMC9715664; doi:10.1038/s41598-022-24788-0)
Supplement: Supplementary file 4 — Supplementary Information 4. [file 41598_2022_24788_MOESM4_ESM.pdf]

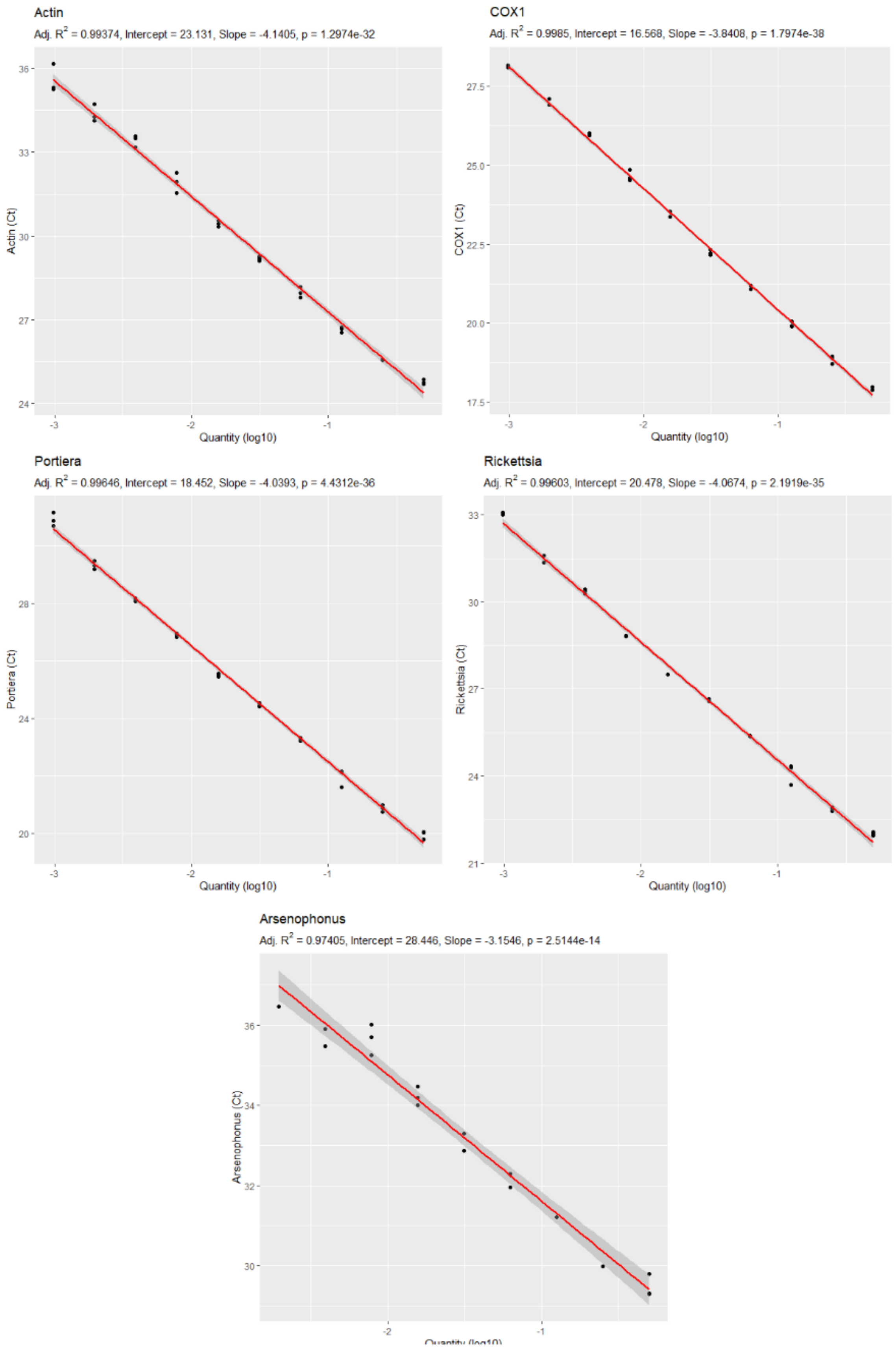

Figure S1 Fitted standard curves used for relative quantification of whitefly endosymbionts, nuclear, and mitochondrial DNA content in qPCR assays along with their corresponding slopes, intercepts, R^2, and p-values.

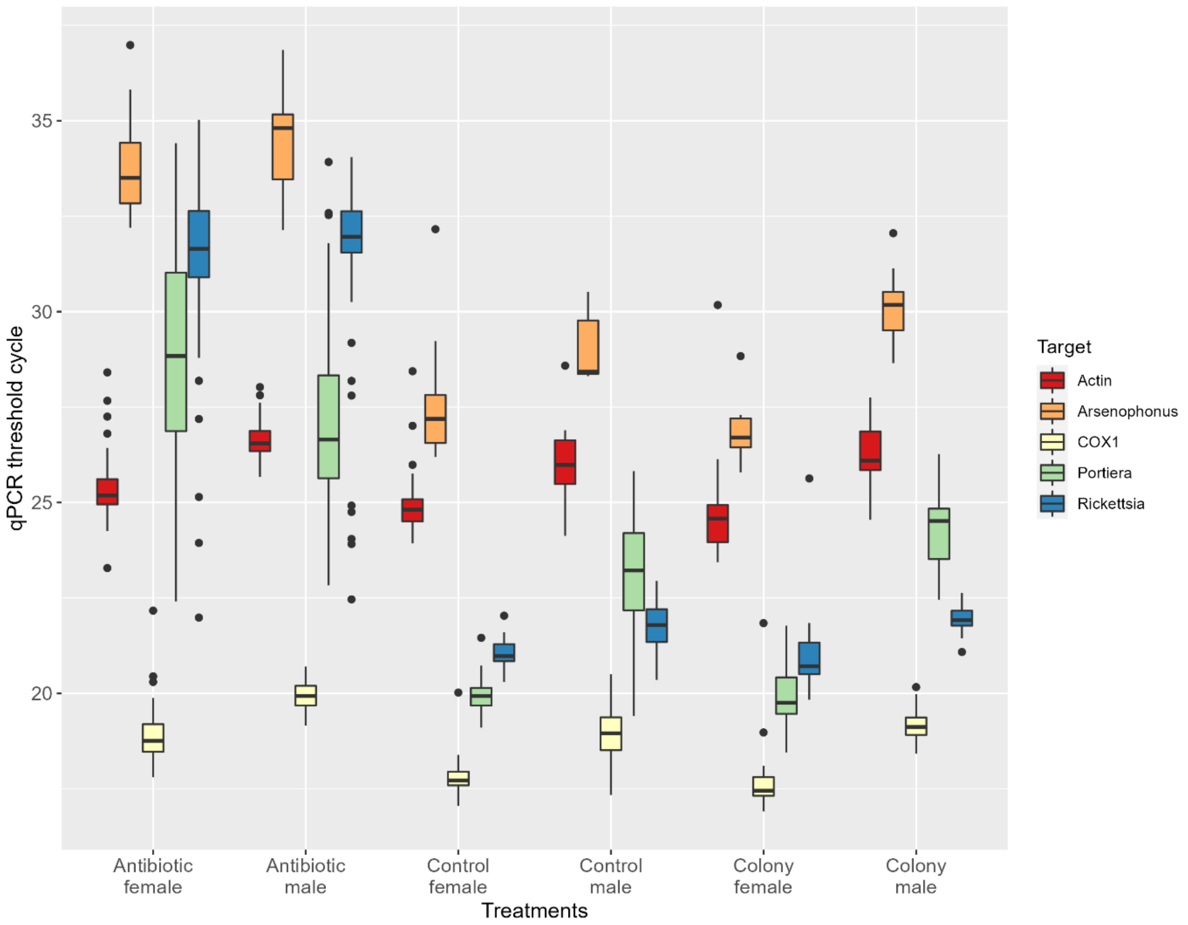

Figure S2. Raw Ct values for all samples across the treatment groups. Boxes represent the data between 25th and 75th percentile, horizontal line within the box represents median, and dots represent the outliers. A data point was considered an outlier as per the standard definition of the R package ggplot2 (when the data point (x) is either lower than Q1 - 1.5 * IQR (interquartile range) or greater than Q3 + 1.5 * IQR). Vertical lines extending the boxes (whiskers) are drawn using standard ggplot2 function and are calculated using the following formulas: upper whisker = min(max(x), Q3 + 1.5 * IQR), lower whisker = max(min(x), Q1 - 1.5 * IQR). IQR = Q3 - Q1. Quantiles are calculated according to the default type 7 definition in R.

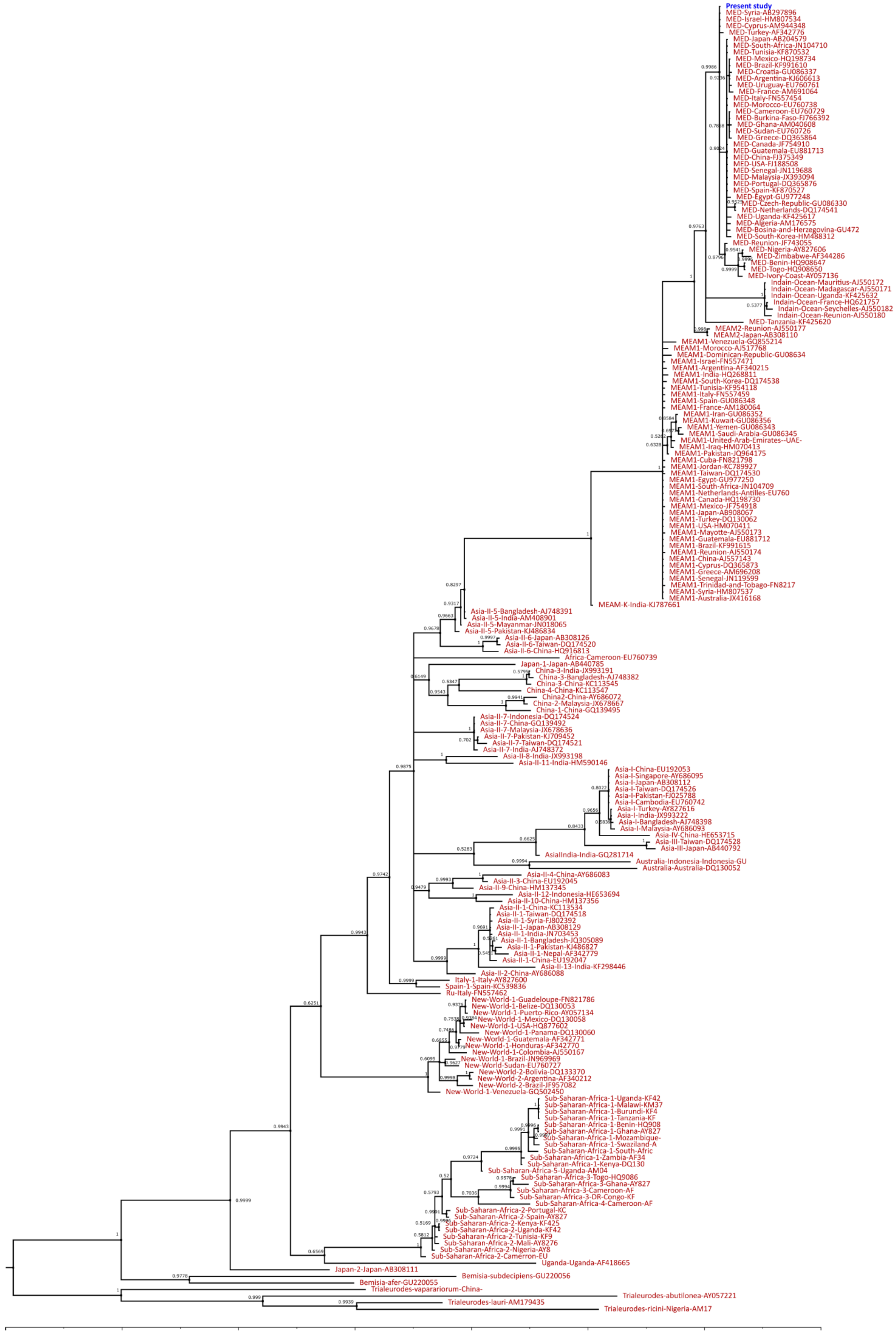

Figure S3: Phylogenetic tree of *Bemisia tabaci* reference mtCOI sequences (red) and the sequence from the present study (blue).

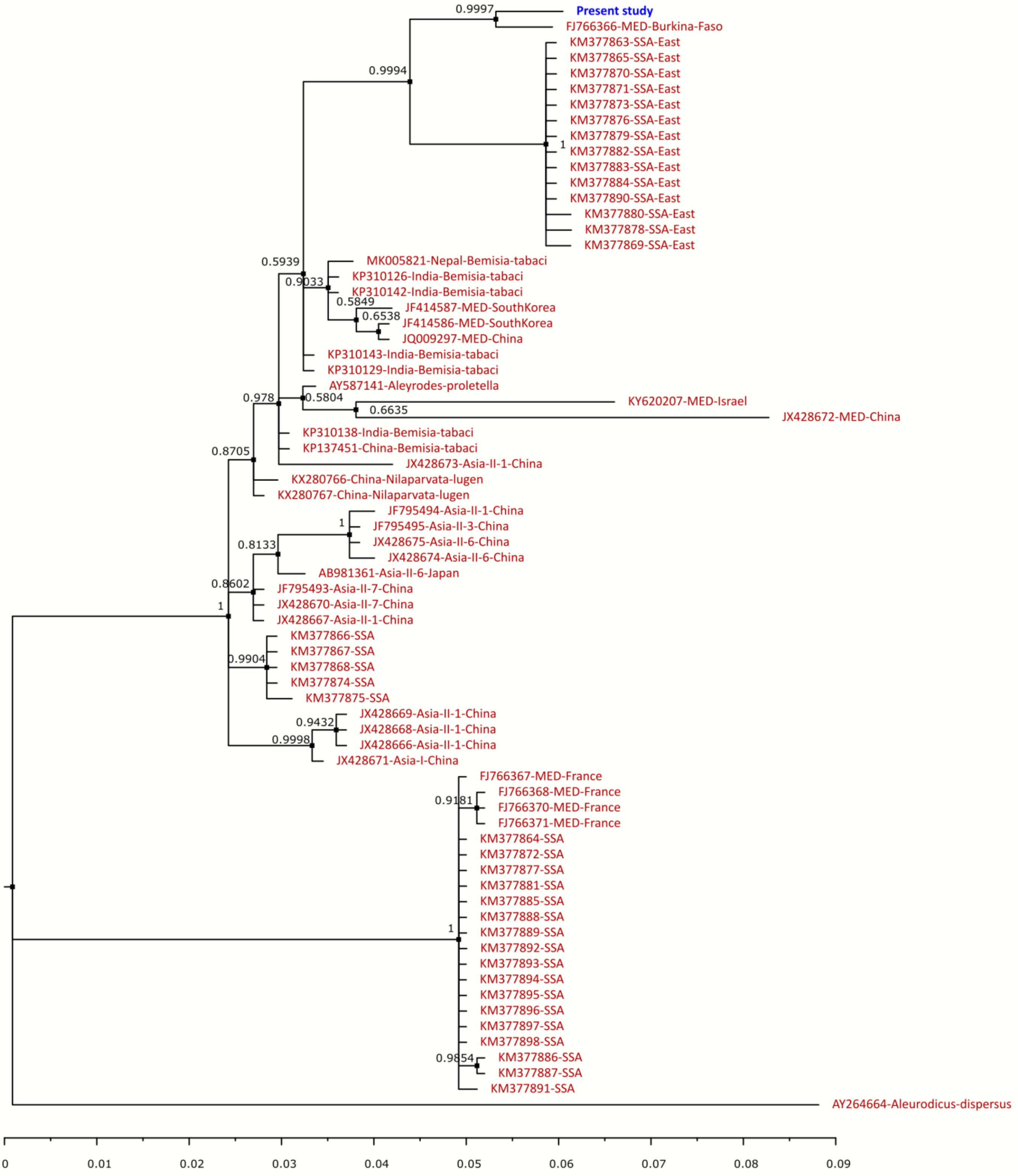

Figure S4: Phylogenetic tree of reference (red) Arsenophonus sequences from Kanakala and Ghanim[11] and top BLAST hits, and the present study (blue) based on partial 23S ribosomal RNA sequence.

Table S1: Counts of Arsenophonus positive and negative samples across treatment groups

Treatment	Positive	Negative	Total	Negative (%)
Antibiotic female	29	19	48	39.6%
Colony female	16	8	24	33.3%
Control female	17	7	24	29.2%
Antibiotic male	17	31	48	64.6%
Control male	5	11	16	68.8%
Colony male	12	12	24	50.0%

Table S2: Results of Fisher's exact test for Arsenophonus presence

Comparison	p.Fisher	p.adj.Fishe	Cramer.V
Antibiotic female : Antibiotic male	0.0241	0.3210	0.2500
Antibiotic female : Control female	0.4440	1.0000	0.1020
Antibiotic female : Control male	0.0808	0.8080	0.2530
Antibiotic female : Colony female	0.7970	1.0000	0.0609
Antibiotic female : Colony male	0.4550	1.0000	0.0992
Antibiotic male : Control female	0.0061	0.0909	0.3340
Antibiotic male : Control male	1.0000	1.0000	0.0380
Antibiotic male : Colony female	0.0229	0.3210	0.2960
Antibiotic male : Colony male	0.3090	1.0000	0.1400
Control female : Control male	0.0230	0.3210	0.3900
Control female : Colony female	1.0000	1.0000	0.0449
Control female : Colony male	0.2380	1.0000	0.2130
Control male : Colony female	0.0515	0.5660	0.3470
Control male : Colony male	0.3320	1.0000	0.1860
Colony female : Colony male	0.3800	1.0000	0.1690

```
################ End of the R Script for plotting raw CT values ################

library(ggplot2)
library(cowplot)
library(tidyr)
library(plyr)
library(multcompView)
library(MASS)
library(readr)
library(naniar)

## locate and read data
raw_data_sample <- read_csv("Data-CT_Plot.csv")
#View(raw_data_sample)


#Clean up the data
raw_data_sample$Group <- gsub('-','_', raw_data_sample$Group)
raw_data_sample <- subset(raw_data_sample, Group != "NC" & Group != "PC_D1" & Group != "PC_D2" & Group != "PC_D3")
raw_data_sample <- raw_data_sample %>% replace_with_na(replace = list(Ct=40.00000))

p<-ggplot(raw_data_sample, aes(x=Group, y=Ct, fill=Target)) + scale_fill_manual(values=c("#d7191c","#fdae61","#ffffbf","#abdda4","#2b83ba")) +
  geom_boxplot(show.legend = TRUE) +
  #ggtitle("Actin", subtitle = "Not normalized, transformed") +
  xlab("Treatments") + ylab("qPCR threshold cycle") +
  scale_x_discrete(labels = str_wrap(c("Antibiotic female", "Antibiotic male", "Control female", "Control male", "Colony female", "Colony male"), width = 10)) +
  theme(axis.text = element_text(size = 11)) +
  theme(axis.title = element_text(size = 12))
p
ggsave(filename = "Raw-Ct.tiff", path="Paper/Graphs", width = 238, height = 185, units="mm", device='tiff', dpi=300, compression = "lzw")

################ Beginning of the R Script for plotting raw CT values ################
```

```r
################ Beginning of the R Script for Actin data analysis ################

library(ggplot2)
library(cowplot)
library(tidyr)
library(plyr)
library(multcompView)
library(MASS)
library(readr)
library(naniar)
library(gridExtra)

## locate and read data
raw_data_sample <- read_csv("Data_samples.csv")
#View(raw_data_sample)
raw_data_curves <- read_csv("Data_curves.csv")
#View(raw_data_curves)

#Clean up the data
raw_data_sample$Group <- gsub('-','_', raw_data_sample$Group)
raw_data_sample <- subset(raw_data_sample, Group != "NC" & Group != "PC_D1" & Group != "PC_D2" & Group != "PC_D3")

raw_data_sample <- raw_data_sample %>% replace_with_na(replace = list(Actin=40.00000))


#Function for plotting standard curves
ggplotRegression <- function (fit) {

  require(ggplot2)

  ggplot(fit$model, aes_string(x = names(fit$model)[2], y = names(fit$model)[1])) +
    geom_point() +
    stat_smooth(method = "lm", col = "red") +
    ggtitle(paste("Standard curve for", names(fit$model)[1]),
            subtitle = substitute(paste("Adj. R"^2," = ",r,
                                  ", Intercept = ",i,
                                  ", Slope = ",s,
                                  ", p = ",p),
                                  list(r= signif(summary(fit)$adj.r.squared, 5),i= signif(fit$coef[[1]],5 ),s=signif(fit$coef[[2]], 5), p=signif(summary(fit)$coef[2,4], 5))
                                 )
           ) + xlab(paste(names(fit$model)[2], "(log10)")) + ylab(paste(names(fit$model)[1], "(Ct)"))
}


#Make standard curves, save models to "curves" and parameters to "curves_param", and plot the curves
curves_param <- data.frame()
curves <- list()
raw_data_curves_trans <- raw_data_curves
raw_data_curves_trans[, "Quantity"] = log10(raw_data_curves_trans[, "Quantity"])
for(i in names(raw_data_curves[, -c(2, 3)])[-1]){
  curves[[i]] <- lm(paste(i, " ~ Quantity"), raw_data_curves_trans[, -c(2, 3)])
  tmp <- data.frame(lapply(curves[[i]][["coefficients"]][1:2], type.convert), stringsAsFactors=FALSE)
  tmp[, "rsquared"] = signif(summary(curves[[i]])$adj.r.squared, 5)
  tmp[, "p"] = signif(summary(curves[[i]])$coef[2,4], 5)
  names(tmp)[1] <- "intercept"
  names(tmp)[2] <- "slope"
  curves_param <-  rbind(curves_param, cbind(Gene = i, tmp))
  rm(tmp)
  print(ggplotRegression(curves[[i]]))
  }
rm(raw_data_curves_trans)

pcr_amount <- function(vec, a, b) {
  res <- 10 ^ ((vec - a)/b)
  return(res)
}

```

```r
get_amounts <- function(df, intercept, slope) {

  amounts <- mapply(function(d, a, b) pcr_amount(d, a, b),
                    d = df, a = intercept, b = slope)
  amounts <- as.data.frame(amounts)

  return(amounts)
}


relative_quantities_perSample <- get_amounts(
  raw_data_sample[3:7],
  intercept = curves_param$intercept,
  slope = curves_param$slope
)

not_normalized_perSample <- relative_quantities_perSample
not_normalized_perSample <- cbind(not_normalized_perSample, raw_data_sample[c("ID", "Group")])

powerTransform <- function(y, lambda1, lambda2 = NULL, method = "boxcox") {

  boxcoxTrans <- function(x, lam1, lam2 = NULL) {

    # if we set lambda2 to zero, it becomes the one parameter transformation
    lam2 <- ifelse(is.null(lam2), 0, lam2)

    if (lam1 == 0L) {
      log(y + lam2)
    } else {
      (((y + lam2)^lam1) - 1) / lam1
    }
  }

  switch(method
         , boxcox = boxcoxTrans(y, lambda1, lambda2)
         , tukey = y^lambda1
  )
}

# run the box-cox transformation to determine optimal lambda
bc_nn <- boxcox(not_normalized_perSample$Actin ~ not_normalized_perSample$Group)

#save and print lambda
(lambda_nn <- bc_nn$x[which.max(bc_nn$y)])

#BoxCox transform the data
not_normalized_perSample <- mutate(not_normalized_perSample, bcActin = powerTransform(not_normalized_perSample$Actin, lambda_nn))

#ANOVA model using NOT normalized, transformed data
ANOVA_model_nn <- aov(not_normalized_perSample$bcActin~Group, data=not_normalized_perSample)
par(mfrow = c(2, 2))
plot(ANOVA_model_nn)
summary(ANOVA_model_nn)
ANOVA_model_nn

#Post-hoc Tukey test
tHSD_nn <- TukeyHSD(ANOVA_model_nn, ordered = FALSE, conf.level = 0.95)
tHSD_nn

#Function to generate letter groupings for graphing
generate_label_df <- function(d, HSD, flev, colNo){
  # Extract labels and factor levels from Tukey post-hoc
  Tukey.levels <- HSD[[flev]][,4]
  Tukey.labels <- multcompLetters(Tukey.levels)['Letters']
  plot.labels <- names(Tukey.labels[['Letters']])

  # Get highest quantile for Tukey's 5 number summary and add a bit of space to buffer between
  # upper quantile and label placement
  names(d)[colNo] <- "transformed"
```

```r
  boxplot.df <- ddply(d, flev, function (x) max(fivenum(x$transformed)) + 0.2)

  # Create a data frame out of the factor levels and Tukey's homogenous group letters
  plot.levels <- data.frame(plot.labels, labels = Tukey.labels[['Letters']],
                            stringsAsFactors = FALSE)

  # Merge it with the labels
  labels.df <- merge(plot.levels, boxplot.df, by.x = 'plot.labels', by.y = flev, sort = FALSE)

  return(labels.df)
}

not_normalized_perSample <- mutate(not_normalized_perSample, OsActin = not_normalized_perSample$Actin)

library(tidyverse)
not_normalized_perSample <- not_normalized_perSample %>%
  mutate(sex = ifelse(grepl("M$", Group), "Male", "Female"))


#Box plot NOT normalized data with Tukey letters, on transformed scale
p3<-ggplot(not_normalized_perSample, aes(x=Group, y=bcActin)) +
  geom_boxplot(show.legend = FALSE , aes(fill=sex)) + scale_fill_manual(values=c("#bdbdbd", "#636363")) +
  geom_text(data = generate_label_df(not_normalized_perSample, tHSD_nn, 'Group', 8), aes(x = plot.labels, y = V1, label = labels)) +
  ggtitle("Actin", subtitle = paste("Not normalized, transformed scale, Box Cox lambda = ", round(lambda_nn, digits=3))) +
  xlab("Treatments") + ylab("Relative quantity (transformed)") +
  theme(axis.text = element_text(size = 11)) +
  theme(axis.title = element_text(size = 12)) +
  theme(plot.title = element_text(face="bold")) +
  scale_x_discrete(labels = str_wrap(c("Antibiotic female", "Antibiotic male", "Control female", "Control male", "Colony female", "Colony male"), width = 10))
p3
ggsave(filename = "Actin-NN-Ts.tiff", path="Paper/Graphs", width = 185, height = 185, units="mm", device='tiff', dpi=300, compression = "lzw")



#Box plot NOT normalized data with Tukey letters, on original scale
p4<-ggplot(not_normalized_perSample, aes(x=Group, y=OsActin)) +
  geom_boxplot(show.legend = FALSE , aes(fill=sex)) + scale_fill_manual(values=c("#bdbdbd", "#636363")) +
  geom_text(data = generate_label_df(not_normalized_perSample, tHSD_nn, 'Group', 9), aes(x = plot.labels, y = V1, label = labels)) +
  ggtitle("Actin", subtitle = paste("Not normalized, original scale, Box Cox lambda = ", round(lambda_nn, digits=3))) +
  xlab("Treatments") + ylab("Relative quantity") +
  theme(axis.text = element_text(size = 11)) +
  theme(axis.title = element_text(size = 12)) +
  theme(plot.title = element_text(face="bold")) +
  scale_x_discrete(labels = str_wrap(c("Antibiotic female", "Antibiotic male", "Control female", "Control male", "Colony female", "Colony male"), width = 10))
p4
ggsave(filename = "Actin-NN-Os.tiff", path="Paper/Graphs", width = 185, height = 185, units="mm", device='tiff', dpi=300, compression = "lzw")

################ End of the R Script for Actin data analysis ################
```

```r
################ Beginning of the R Script for COX1 data analysis ################

library(ggplot2)
library(cowplot)
library(tidyr)
library(plyr)
library(multcompView)
library(MASS)
library(readr)
library(naniar)
library(gridExtra)

## locate and read data
raw_data_sample <- read_csv("Data_samples.csv")
#View(raw_data_sample)
raw_data_curves <- read_csv("Data_curves.csv")
#View(raw_data_curves)

#Clean up the data
raw_data_sample$Group <- gsub('-','_', raw_data_sample$Group)
raw_data_sample <- subset(raw_data_sample, Group != "NC" & Group != "PC_D1" & Group != "PC_D2" & Group != "PC_D3")

raw_data_sample <- raw_data_sample %>% replace_with_na(replace = list(COX1=40.00000))


#Function for plotting standard curves
ggplotRegression <- function (fit) {

  require(ggplot2)

  ggplot(fit$model, aes_string(x = names(fit$model)[2], y = names(fit$model)[1])) +
    geom_point() +
    stat_smooth(method = "lm", col = "red") +
    ggtitle(paste("Standard curve for", names(fit$model)[1]),
            subtitle = substitute(paste("Adj. R"^2," = ",r,
                                 ", Intercept = ",i,
                                 ", Slope = ",s,
                                 ", p = ",p),
                                 list(r= signif(summary(fit)$adj.r.squared, 5),i= signif(fit$coef[[1]],5 ),s=signif(fit$coef[[2]], 5), p=signif(summary(fit)$coef[2,4], 5))
                                )
           ) + xlab(paste(names(fit$model)[2], "(log10)")) + ylab(paste(names(fit$model)[1], "(Ct)"))
}


#Make standard curves, save models to "curves" and parameters to "curves_param", and plot the curves
curves_param <- data.frame()
curves <- list()
raw_data_curves_trans <- raw_data_curves
raw_data_curves_trans[, "Quantity"] = log10(raw_data_curves_trans[, "Quantity"])
for(i in names(raw_data_curves[, -c(2, 3)])[-1]){
  curves[[i]] <- lm(paste(i, " ~ Quantity"), raw_data_curves_trans[, -c(2, 3)])
  tmp <- data.frame(lapply(curves[[i]][["coefficients"]][1:2], type.convert), stringsAsFactors=FALSE)
  tmp[, "rsquared"] = signif(summary(curves[[i]])$adj.r.squared, 5)
  tmp[, "p"] = signif(summary(curves[[i]])$coef[2,4], 5)
  names(tmp)[1] <- "intercept"
  names(tmp)[2] <- "slope"
  curves_param <-  rbind(curves_param, cbind(Gene = i, tmp))
  rm(tmp)
  print(ggplotRegression(curves[[i]]))
  }
rm(raw_data_curves_trans)

pcr_amount <- function(vec, a, b) {
  res <- 10 ^ ((vec - a)/b)
  return(res)
}

```

```r
get_amounts <- function(df, intercept, slope) {

  amounts <- mapply(function(d, a, b) pcr_amount(d, a, b),
                    d = df, a = intercept, b = slope)
  amounts <- as.data.frame(amounts)

  return(amounts)
}


pcr_normalize <- function(vec, ref, mode) {
  if (mode == 'subtract') {
    res <- vec - ref
  } else if (mode == 'divide') {
    res <- vec / ref
  } else {
    stop("mode can be one of 'subtract' or 'divide'.")
  }
  return(res)
}


relative_quantities_perSample <- get_amounts(
  raw_data_sample[3:7],
  intercept = curves_param$intercept,
  slope = curves_param$slope
)

normalized_perSample <- pcr_normalize(relative_quantities_perSample, relative_quantities_perSample$Actin, mode = 'divide')
normalized_perSample <- cbind(normalized_perSample, raw_data_sample[c("ID", "Group")])

not_normalized_perSample <- relative_quantities_perSample
not_normalized_perSample <- cbind(not_normalized_perSample, raw_data_sample[c("ID", "Group")])

#Comment next line to apply normalization
#normalized_perSample <- not_normalized_perSample

powerTransform <- function(y, lambda1, lambda2 = NULL, method = "boxcox") {

  boxcoxTrans <- function(x, lam1, lam2 = NULL) {

    # if we set lambda2 to zero, it becomes the one parameter transformation
    lam2 <- ifelse(is.null(lam2), 0, lam2)

    if (lam1 == 0L) {
      log(y + lam2)
    } else {
      (((y + lam2)^lam1) - 1) / lam1
    }
  }

  switch(method
         , boxcox = boxcoxTrans(y, lambda1, lambda2)
         , tukey = y^lambda1
  )
}

# run the box-cox transformation to determine optimal lambda
bc <- boxcox(normalized_perSample$COX1 ~ normalized_perSample$Group)
bc_nn <- boxcox(not_normalized_perSample$COX1 ~ not_normalized_perSample$Group)

#save and print lambda
(lambda <- bc$x[which.max(bc$y)])
(lambda_nn <- bc_nn$x[which.max(bc_nn$y)])

#BoxCox transform the data
normalized_perSample <- mutate(normalized_perSample, bcCOX1 = powerTransform(normalized_perSample$COX1, lambda))
not_normalized_perSample <- mutate(not_normalized_perSample, bcCOX1 = powerTransform(not_normalized_perSample$COX1, lambda_nn))
```

```r
#ANOVA model using normalized, transformed data
ANOVA_model_norm <- aov(normalized_perSample$bcCOX1~Group, data=normalized_perSample)
par(mfrow = c(2, 2))
plot(ANOVA_model_norm)
summary(ANOVA_model_norm)
ANOVA_model_norm

#ANOVA model using NOT normalized, transformed data
ANOVA_model_nn <- aov(not_normalized_perSample$bcCOX1~Group, data=not_normalized_perSample)
par(mfrow = c(2, 2))
plot(ANOVA_model_nn)
summary(ANOVA_model_nn)
ANOVA_model_nn

#Post-hoc Tukey test
tHSD <- TukeyHSD(ANOVA_model_norm, ordered = FALSE, conf.level = 0.95)
tHSD
tHSD_nn <- TukeyHSD(ANOVA_model_nn, ordered = FALSE, conf.level = 0.95)
tHSD_nn

#Function to generate letter groupings for graphing
generate_label_df <- function(d, HSD, flev, colNo){
  # Extract labels and factor levels from Tukey post-hoc
  Tukey.levels <- HSD[[flev]][,4]
  Tukey.labels <- multcompLetters(Tukey.levels)['Letters']
  plot.labels <- names(Tukey.labels[['Letters']])

  # Get highest quantile for Tukey's 5 number summary and add a bit of space to buffer between
  # upper quantile and label placement
  names(d)[colNo] <- "transformed"
  boxplot.df <- ddply(d, flev, function (x) max(fivenum(x$transformed)) + 0.2)

  # Create a data frame out of the factor levels and Tukey's homogenous group letters
  plot.levels <- data.frame(plot.labels, labels = Tukey.labels[['Letters']],
                            stringsAsFactors = FALSE)

  # Merge it with the labels
  labels.df <- merge(plot.levels, boxplot.df, by.x = 'plot.labels', by.y = flev, sort = FALSE)

  return(labels.df)
}

normalized_perSample <- mutate(normalized_perSample, OsCOX1 = normalized_perSample$COX1)
not_normalized_perSample <- mutate(not_normalized_perSample, OsCOX1 = not_normalized_perSample$COX1)

library(tidyverse)
not_normalized_perSample <- not_normalized_perSample %>%
  mutate(sex = ifelse(grepl("M$", Group), "Male", "Female"))

normalized_perSample <- normalized_perSample %>%
  mutate(sex = ifelse(grepl("M$", Group), "Male", "Female"))

#Box plot Actin normalized data with Tukey letters, on transformed scale
p1<-ggplot(normalized_perSample, aes(x=Group, y=bcCOX1)) +
  geom_boxplot(show.legend = FALSE , aes(fill=sex)) + scale_fill_manual(values=c("#bdbdbd", "#636363")) +
  geom_text(data = generate_label_df(normalized_perSample, tHSD, 'Group', 8), aes(x = plot.labels, y = V1, label = labels)) +
  ggtitle("COX1", subtitle = paste("Actin normalized, transformed scale, Box Cox lambda = ", round(lambda, digits=3))) +
  xlab("Treatments") + ylab("Relative quantity (transformed)") +
  theme(axis.text = element_text(size = 11)) +
  theme(axis.title = element_text(size = 12)) +
  theme(plot.title = element_text(face="bold")) +
  scale_x_discrete(labels = str_wrap(c("Antibiotic female", "Antibiotic male", "Control female", "Control male", "Colony female", "Colony male"), width = 10))
p1
#ggsave(filename = "COX1-AN-Ts.tiff", path="Paper/original scale", width = 600,
```

```r
height = 700, units="px", device='tiff', dpi=96)


#Box plot Actin normalized data with Tukey letters, on original scale
p2<-ggplot(normalized_perSample, aes(x=Group, y=OsCOX1)) +
  geom_boxplot(show.legend = FALSE , aes(fill=sex)) + scale_fill_manual(values=c("#bdbdbd", "#636363")) +
  geom_text(data = generate_label_df(normalized_perSample, tHSD, 'Group', 9), aes(x = plot.labels, y = V1, label = labels)) +
  ggtitle("COX1", subtitle = paste("Actin normalized, original scale, Box Cox lambda = ", round(lambda, digits=3))) +
  xlab("Treatments") + ylab("Relative quantity") +
  theme(axis.text = element_text(size = 11)) +
  theme(axis.title = element_text(size = 12)) +
  theme(plot.title = element_text(face="bold")) +
  scale_x_discrete(labels = str_wrap(c("Antibiotic female", "Antibiotic male", "Control female", "Control male", "Colony female", "Colony male"), width = 10))
p2
#ggsave(filename = "COX1-AN-Os.tiff", path="Paper/original scale", width = 600, height = 700, units="px", device='tiff', dpi=96)


#Box plot NOT normalized data with Tukey letters, on transformed scale
p3<-ggplot(not_normalized_perSample, aes(x=Group, y=bcCOX1)) +
  geom_boxplot(show.legend = FALSE , aes(fill=sex)) + scale_fill_manual(values=c("#bdbdbd", "#636363")) +
  geom_text(data = generate_label_df(not_normalized_perSample, tHSD_nn, 'Group', 8), aes(x = plot.labels, y = V1, label = labels)) +
  ggtitle("COX1", subtitle = paste("Not normalized, transformed scale, Box Cox lambda = ", round(lambda_nn, digits=3))) +
  xlab("Treatments") + ylab("Relative quantity (transformed)") +
  theme(axis.text = element_text(size = 11)) +
  theme(axis.title = element_text(size = 12)) +
  theme(plot.title = element_text(face="bold")) +
  scale_x_discrete(labels = str_wrap(c("Antibiotic female", "Antibiotic male", "Control female", "Control male", "Colony female", "Colony male"), width = 10))
p3
#ggsave(filename = "COX1-NN-Ts.tiff", path="Paper/original scale", width = 600, height = 700, units="px", device='tiff', dpi=96)


#Box plot NOT normalized data with Tukey letters, on original scale
p4<-ggplot(not_normalized_perSample, aes(x=Group, y=OsCOX1)) +
  geom_boxplot(show.legend = FALSE , aes(fill=sex)) + scale_fill_manual(values=c("#bdbdbd", "#636363")) +
  geom_text(data = generate_label_df(not_normalized_perSample, tHSD_nn, 'Group', 9), aes(x = plot.labels, y = V1, label = labels)) +
  ggtitle("COX1", subtitle = paste("Not normalized, original scale, Box Cox lambda = ", round(lambda_nn, digits=3))) +
  xlab("Treatments") + ylab("Relative quantity") +
  theme(axis.text = element_text(size = 11)) +
  theme(axis.title = element_text(size = 12)) +
  theme(plot.title = element_text(face="bold")) +
  scale_x_discrete(labels = str_wrap(c("Antibiotic female", "Antibiotic male", "Control female", "Control male", "Colony female", "Colony male"), width = 10))
p4
#ggsave(filename = "COX1-NN-Os.tiff", path="Paper/original scale", width = 600, height = 700, units="px", device='tiff', dpi=96)


ggsave(filename = "COX1-Ts.tiff", path="Paper/Graphs", arrangeGrob(p3, p1,nrow = 1), width = 322, height = 185, units="mm", device='tiff', dpi=300, compression = "lzw")
ggsave(filename = "COX1-Os.tiff", path="Paper/Graphs", arrangeGrob(p4, p2,nrow = 1), width = 322, height = 185, units="mm", device='tiff', dpi=300, compression = "lzw")

################ End of the R Script for COX1 data analysis ################
```

```r
################ Beginning of the R Script for Portiera data analysis ################

library(ggplot2)
library(cowplot)
library(tidyr)
library(plyr)
library(multcompView)
library(MASS)
library(readr)
library(naniar)
library(gridExtra)

## locate and read data
raw_data_sample <- read_csv("Data_samples.csv")
#View(raw_data_sample)
raw_data_curves <- read_csv("Data_curves.csv")
#View(raw_data_curves)

#Clean up the data
raw_data_sample$Group <- gsub('-','_', raw_data_sample$Group)
raw_data_sample <- subset(raw_data_sample, Group != "NC" & Group != "PC_D1" & Group != "PC_D2" & Group != "PC_D3")

raw_data_sample <- raw_data_sample %>% replace_with_na(replace = list(Portiera= 40.00000))
raw_data_sample <- raw_data_sample %>% replace_with_na(replace = list(Portiera= 40.00000))



#Function for plotting standard curves
ggplotRegression <- function (fit) {

  require(ggplot2)

  ggplot(fit$model, aes_string(x = names(fit$model)[2], y = names(fit$model)[1])) +
    geom_point() +
    stat_smooth(method = "lm", col = "red") +
    ggtitle(paste("Standard curve for", names(fit$model)[1]),
            subtitle = substitute(paste("Adj. R"^2," = ",r,
                                  ", Intercept = ",i,
                                  ", Slope = ",s,
                                  ", p = ",p),
                                  list(r= signif(summary(fit)$adj.r.squared, 5),i= signif(fit$coef[[1]],5 ),s=signif(fit$coef[[2]], 5), p=signif(summary(fit)$coef[2,4], 5))
                                 )
           ) + xlab(paste(names(fit$model)[2], "(log10)")) + ylab(paste(names(fit$model)[1], "(Ct)"))
}


#Make standard curves, save models to "curves" and parameters to "curves_param", and plot the curves
curves_param <- data.frame()
curves <- list()
raw_data_curves_trans <- raw_data_curves
raw_data_curves_trans[, "Quantity"] = log10(raw_data_curves_trans[, "Quantity"])
for(i in names(raw_data_curves[, -c(2, 3)])[-1]){
  curves[[i]] <- lm(paste(i, " ~ Quantity"), raw_data_curves_trans[, -c(2, 3)])
  tmp <- data.frame(lapply(curves[[i]][["coefficients"]][1:2], type.convert), stringsAsFactors=FALSE)
  tmp[, "rsquared"] = signif(summary(curves[[i]])$adj.r.squared, 5)
  tmp[, "p"] = signif(summary(curves[[i]])$coef[2,4], 5)
  names(tmp)[1] <- "intercept"
  names(tmp)[2] <- "slope"
  curves_param <-  rbind(curves_param, cbind(Gene = i, tmp))
  rm(tmp)
  print(ggplotRegression(curves[[i]]))
  }
rm(raw_data_curves_trans)

pcr_amount <- function(vec, a, b) {
```

```
  res <- 10 ^ ((vec - a)/b)
  return(res)
}

get_amounts <- function(df, intercept, slope) {

  amounts <- mapply(function(d, a, b) pcr_amount(d, a, b),
                    d = df, a = intercept, b = slope)
  amounts <- as.data.frame(amounts)

  return(amounts)
}


pcr_normalize <- function(vec, ref, mode) {
  if (mode == 'subtract') {
    res <- vec - ref
  } else if (mode == 'divide') {
    res <- vec / ref
  } else {
    stop("mode can be one of 'subtract' or 'divide'.")
  }
  return(res)
}


relative_quantities_perSample <- get_amounts(
  raw_data_sample[3:7],
  intercept = curves_param$intercept,
  slope = curves_param$slope
)

normalized_perSample <- pcr_normalize(relative_quantities_perSample, relative_quantities_perSample$Actin, mode = 'divide')
normalized_perSample <- cbind(normalized_perSample, raw_data_sample[c("ID", "Group")])

not_normalized_perSample <- relative_quantities_perSample
not_normalized_perSample <- cbind(not_normalized_perSample, raw_data_sample[c("ID", "Group")])

#Comment next line to apply normalization
#normalized_perSample <- not_normalized_perSample

powerTransform <- function(y, lambda1, lambda2 = NULL, method = "boxcox") {

  boxcoxTrans <- function(x, lam1, lam2 = NULL) {

    # if we set lambda2 to zero, it becomes the one parameter transformation
    lam2 <- ifelse(is.null(lam2), 0, lam2)

    if (lam1 == 0L) {
      log(y + lam2)
    } else {
      (((y + lam2)^lam1) - 1) / lam1
    }
  }

  switch(method
         , boxcox = boxcoxTrans(y, lambda1, lambda2)
         , tukey = y^lambda1
  )
}

# run the box-cox transformation to determine optimal lambda
bc <- boxcox(normalized_perSample$Portiera ~ normalized_perSample$Group)
bc_nn <- boxcox(not_normalized_perSample$Portiera ~ not_normalized_perSample$Group)

#save and print lambda
(lambda <- bc$x[which.max(bc$y)])
(lambda_nn <- bc_nn$x[which.max(bc_nn$y)])

#BoxCox transform the data
normalized_perSample <- mutate(normalized_perSample, bcPortiera = powerTransform(
```

```
normalized_perSample$Portiera, lambda))
not_normalized_perSample <- mutate(not_normalized_perSample, bcPortiera = powerTransform(not_normalized_perSample$Portiera, lambda_nn))

#ANOVA model using normalized, transformed data
ANOVA_model_norm <- aov(normalized_perSample$bcPortiera~Group, data= normalized_perSample)
par(mfrow = c(2, 2))
plot(ANOVA_model_norm)
summary(ANOVA_model_norm)
ANOVA_model_norm

#ANOVA model using NOT normalized, transformed data
ANOVA_model_nn <- aov(not_normalized_perSample$bcPortiera~Group, data= not_normalized_perSample)
par(mfrow = c(2, 2))
plot(ANOVA_model_nn)
summary(ANOVA_model_nn)
ANOVA_model_nn

#Post-hoc Tukey test
tHSD <- TukeyHSD(ANOVA_model_norm, ordered = FALSE, conf.level = 0.95)
tHSD
tHSD_nn <- TukeyHSD(ANOVA_model_nn, ordered = FALSE, conf.level = 0.95)
tHSD_nn

#Function to generate letter groupings for graphing
generate_label_df <- function(d, HSD, flev, colNo){
  # Extract labels and factor levels from Tukey post-hoc
  Tukey.levels <- HSD[[flev]][,4]
  Tukey.labels <- multcompLetters(Tukey.levels)['Letters']
  plot.labels <- names(Tukey.labels[['Letters']])

  # Get highest quantile for Tukey's 5 number summary and add a bit of space to buffer between
  # upper quantile and label placement
  names(d)[colNo] <- "transformed"
  boxplot.df <- ddply(d, flev, function (x) max(fivenum(x$transformed)) + 0.2)

  # Create a data frame out of the factor levels and Tukey's homogenous group letters
  plot.levels <- data.frame(plot.labels, labels = Tukey.labels[['Letters']],
                            stringsAsFactors = FALSE)

  # Merge it with the labels
  labels.df <- merge(plot.levels, boxplot.df, by.x = 'plot.labels', by.y = flev, sort = FALSE)

  return(labels.df)
}

normalized_perSample <- mutate(normalized_perSample, OsPortiera = normalized_perSample$Portiera)
not_normalized_perSample <- mutate(not_normalized_perSample, OsPortiera = not_normalized_perSample$Portiera)

library(tidyverse)
not_normalized_perSample <- not_normalized_perSample %>%
  mutate(sex = ifelse(grepl("M$", Group), "Male", "Female"))

normalized_perSample <- normalized_perSample %>%
  mutate(sex = ifelse(grepl("M$", Group), "Male", "Female"))

#Box plot Actin normalized data with Tukey letters, on transformed scale
p1<-ggplot(normalized_perSample, aes(x=Group, y=bcPortiera)) +
  geom_boxplot(show.legend = FALSE , aes(fill=sex)) + scale_fill_manual(values=c("#bdbdbd", "#636363")) +
  geom_text(data = generate_label_df(normalized_perSample, tHSD, 'Group', 8), aes(x = plot.labels, y = V1, label = labels)) +
  ggtitle("Portiera", subtitle = paste("Actin normalized, transformed scale, Box Cox lambda = ", round(lambda, digits=3))) +
  xlab("Treatments") + ylab("Relative quantity (transformed)") +
  theme(axis.text = element_text(size = 11)) +
  theme(axis.title = element_text(size = 12)) +
```

```
  theme(plot.title = element_text(face="bold")) +
  scale_x_discrete(labels = str_wrap(c("Antibiotic female", "Antibiotic male", "Control female", "Control male", "Colony female", "Colony male"), width = 10))
p1
#ggsave(filename = "Portiera-AN-Ts.tiff", path="Paper/original scale", width = 600, height = 700, units="px", device='tiff', dpi=96)


#Box plot Actin normalized data with Tukey letters, on original scale
p2<-ggplot(normalized_perSample, aes(x=Group, y=OsPortiera)) +
  geom_boxplot(show.legend = FALSE , aes(fill=sex)) + scale_fill_manual(values=c("#bdbdbd", "#636363")) +
  geom_text(data = generate_label_df(normalized_perSample, tHSD, 'Group', 9), aes(x = plot.labels, y = V1, label = labels)) +
  ggtitle("Portiera", subtitle = paste("Actin normalized, original scale, Box Cox lambda = ", round(lambda, digits=3))) +
  xlab("Treatments") + ylab("Relative quantity") +
  theme(axis.text = element_text(size = 11)) +
  theme(axis.title = element_text(size = 12)) +
  theme(plot.title = element_text(face="bold")) +
  scale_x_discrete(labels = str_wrap(c("Antibiotic female", "Antibiotic male", "Control female", "Control male", "Colony female", "Colony male"), width = 10))
p2
#ggsave(filename = "Portiera-AN-Os.tiff", path="Paper/original scale", width = 600, height = 700, units="px", device='tiff', dpi=96)


#Box plot NOT normalized data with Tukey letters, on transformed scale
p3<-ggplot(not_normalized_perSample, aes(x=Group, y=bcPortiera)) +
  geom_boxplot(show.legend = FALSE , aes(fill=sex)) + scale_fill_manual(values=c("#bdbdbd", "#636363")) +
  geom_text(data = generate_label_df(not_normalized_perSample, tHSD_nn, 'Group', 8), aes(x = plot.labels, y = V1, label = labels)) +
  ggtitle("Portiera", subtitle = paste("Not normalized, transformed scale, Box Cox lambda = ", round(lambda_nn, digits=3))) +
  xlab("Treatments") + ylab("Relative quantity (transformed)") +
  theme(axis.text = element_text(size = 11)) +
  theme(axis.title = element_text(size = 12)) +
  theme(plot.title = element_text(face="bold")) +
  scale_x_discrete(labels = str_wrap(c("Antibiotic female", "Antibiotic male", "Control female", "Control male", "Colony female", "Colony male"), width = 10))
p3
#ggsave(filename = "Portiera-NN-Ts.tiff", path="Paper/original scale", width = 600, height = 700, units="px", device='tiff', dpi=96)


#Box plot NOT normalized data with Tukey letters, on original scale
p4<-ggplot(not_normalized_perSample, aes(x=Group, y=OsPortiera)) +
  geom_boxplot(show.legend = FALSE , aes(fill=sex)) + scale_fill_manual(values=c("#bdbdbd", "#636363")) +
  geom_text(data = generate_label_df(not_normalized_perSample, tHSD_nn, 'Group', 9), aes(x = plot.labels, y = V1, label = labels)) +
  ggtitle("Portiera", subtitle = paste("Not normalized, original scale, Box Cox lambda = ", round(lambda_nn, digits=3))) +
  xlab("Treatments") + ylab("Relative quantity") +
  theme(axis.text = element_text(size = 11)) +
  theme(axis.title = element_text(size = 12)) +
  theme(plot.title = element_text(face="bold")) +
  scale_x_discrete(labels = str_wrap(c("Antibiotic female", "Antibiotic male", "Control female", "Control male", "Colony female", "Colony male"), width = 10))
p4
#ggsave(filename = "Portiera-NN-Os.tiff", path="Paper/original scale", width = 600, height = 700, units="px", device='tiff', dpi=96)


ggsave(filename = "Portiera-Ts.tiff", path="Paper/Graphs", arrangeGrob(p3, p1,nrow = 1), width = 322, height = 185, units="mm", device='tiff', dpi=300, compression = "lzw")
ggsave(filename = "Portiera-Os.tiff", path="Paper/Graphs", arrangeGrob(p4, p2,nrow = 1), width = 322, height = 185, units="mm", device='tiff', dpi=300, compression = "lzw")

################ End of the R Script for Portiera data analysis ################
```

```r
################ Beginning of the R Script for Rickettsia data analysis ################

library(ggplot2)
library(cowplot)
library(tidyr)
library(plyr)
library(multcompView)
library(MASS)
library(readr)
library(naniar)
library(gridExtra)

## locate and read data
raw_data_sample <- read_csv("Data_samples.csv")
#View(raw_data_sample)
raw_data_curves <- read_csv("Data_curves.csv")
#View(raw_data_curves)

#Clean up the data
raw_data_sample$Group <- gsub('-','_', raw_data_sample$Group)
raw_data_sample <- subset(raw_data_sample, Group != "NC" & Group != "PC_D1" & Group != "PC_D2" & Group != "PC_D3")

raw_data_sample <- raw_data_sample %>% replace_with_na(replace = list(Rickettsia= 40.00000))


#Function for plotting standard curves
ggplotRegression <- function (fit) {

  require(ggplot2)

  ggplot(fit$model, aes_string(x = names(fit$model)[2], y = names(fit$model)[1])) +
    geom_point() +
    stat_smooth(method = "lm", col = "red") +
    ggtitle(paste("Standard curve for", names(fit$model)[1]),
            subtitle = substitute(paste("Adj. R"^2," = ",r,
                                  ", Intercept = ",i,
                                  ", Slope = ",s,
                                  ", p = ",p),
                                  list(r= signif(summary(fit)$adj.r.squared, 5),i= signif(fit$coef[[1]],5 ),s=signif(fit$coef[[2]], 5), p=signif(summary(fit)$coef[2,4], 5))
                                 )
           ) + xlab(paste(names(fit$model)[2], "(log10)")) + ylab(paste(names(fit$model)[1], "(Ct)"))
}


#Make standard curves, save models to "curves" and parameters to "curves_param", and plot the curves
curves_param <- data.frame()
curves <- list()
raw_data_curves_trans <- raw_data_curves
raw_data_curves_trans[, "Quantity"] = log10(raw_data_curves_trans[, "Quantity"])
for(i in names(raw_data_curves[, -c(2, 3)])[-1]){
  curves[[i]] <- lm(paste(i, " ~ Quantity"), raw_data_curves_trans[, -c(2, 3)])
  tmp <- data.frame(lapply(curves[[i]][["coefficients"]][1:2], type.convert), stringsAsFactors=FALSE)
  tmp[, "rsquared"] = signif(summary(curves[[i]])$adj.r.squared, 5)
  tmp[, "p"] = signif(summary(curves[[i]])$coef[2,4], 5)
  names(tmp)[1] <- "intercept"
  names(tmp)[2] <- "slope"
  curves_param <-  rbind(curves_param, cbind(Gene = i, tmp))
  rm(tmp)
  print(ggplotRegression(curves[[i]]))
  }
rm(raw_data_curves_trans)

pcr_amount <- function(vec, a, b) {
  res <- 10 ^ ((vec - a)/b)
  return(res)
```

```
}

get_amounts <- function(df, intercept, slope) {

  amounts <- mapply(function(d, a, b) pcr_amount(d, a, b),
                    d = df, a = intercept, b = slope)
  amounts <- as.data.frame(amounts)

  return(amounts)
}


pcr_normalize <- function(vec, ref, mode) {
  if (mode == 'subtract') {
    res <- vec - ref
  } else if (mode == 'divide') {
    res <- vec / ref
  } else {
    stop("mode can be one of 'subtract' or 'divide'.")
  }
  return(res)
}


relative_quantities_perSample <- get_amounts(
  raw_data_sample[3:7],
  intercept = curves_param$intercept,
  slope = curves_param$slope
)

normalized_perSample <- pcr_normalize(relative_quantities_perSample, relative_quantities_perSample$Actin, mode = 'divide')
normalized_perSample <- cbind(normalized_perSample, raw_data_sample[c("ID", "Group")])

not_normalized_perSample <- relative_quantities_perSample
not_normalized_perSample <- cbind(not_normalized_perSample, raw_data_sample[c("ID", "Group")])

#Comment next line to apply normalization
#normalized_perSample <- not_normalized_perSample

powerTransform <- function(y, lambda1, lambda2 = NULL, method = "boxcox") {

  boxcoxTrans <- function(x, lam1, lam2 = NULL) {

    # if we set lambda2 to zero, it becomes the one parameter transformation
    lam2 <- ifelse(is.null(lam2), 0, lam2)

    if (lam1 == 0L) {
      log(y + lam2)
    } else {
      (((y + lam2)^lam1) - 1) / lam1
    }
  }

  switch(method
         , boxcox = boxcoxTrans(y, lambda1, lambda2)
         , tukey = y^lambda1
  )
}

# run the box-cox transformation to determine optimal lambda
bc <- boxcox(normalized_perSample$Rickettsia ~ normalized_perSample$Group)
bc_nn <- boxcox(not_normalized_perSample$Rickettsia ~ not_normalized_perSample$Group)

#save and print lambda
(lambda <- bc$x[which.max(bc$y)])
(lambda_nn <- bc_nn$x[which.max(bc_nn$y)])

#BoxCox transform the data
normalized_perSample <- mutate(normalized_perSample, bcRickettsia = powerTransform(normalized_perSample$Rickettsia, lambda))
not_normalized_perSample <- mutate(not_normalized_perSample, bcRickettsia =
```

```
powerTransform(not_normalized_perSample$Rickettsia, lambda_nn))

#ANOVA model using normalized, transformed data
ANOVA_model_norm <- aov(normalized_perSample$bcRickettsia~Group, data= normalized_perSample)
par(mfrow = c(2, 2))
plot(ANOVA_model_norm)
summary(ANOVA_model_norm)
ANOVA_model_norm

#ANOVA model using NOT normalized, transformed data
ANOVA_model_nn <- aov(not_normalized_perSample$bcRickettsia~Group, data= not_normalized_perSample)
par(mfrow = c(2, 2))
plot(ANOVA_model_nn)
summary(ANOVA_model_nn)
ANOVA_model_nn

#Post-hoc Tukey test
tHSD <- TukeyHSD(ANOVA_model_norm, ordered = FALSE, conf.level = 0.95)
tHSD
tHSD_nn <- TukeyHSD(ANOVA_model_nn, ordered = FALSE, conf.level = 0.95)
tHSD_nn

#Function to generate letter groupings for graphing
generate_label_df <- function(d, HSD, flev, colNo){
  # Extract labels and factor levels from Tukey post-hoc
  Tukey.levels <- HSD[[flev]][,4]
  Tukey.labels <- multcompLetters(Tukey.levels)['Letters']
  plot.labels <- names(Tukey.labels[['Letters']])

  # Get highest quantile for Tukey's 5 number summary and add a bit of space to buffer between
  # upper quantile and label placement
  names(d)[colNo] <- "transformed"
  boxplot.df <- ddply(d, flev, function (x) max(fivenum(x$transformed)) + 0.2)

  # Create a data frame out of the factor levels and Tukey's homogenous group letters
  plot.levels <- data.frame(plot.labels, labels = Tukey.labels[['Letters']],
                            stringsAsFactors = FALSE)

  # Merge it with the labels
  labels.df <- merge(plot.levels, boxplot.df, by.x = 'plot.labels', by.y = flev, sort = FALSE)

  return(labels.df)
}

normalized_perSample <- mutate(normalized_perSample, OsRickettsia = normalized_perSample$Rickettsia)
not_normalized_perSample <- mutate(not_normalized_perSample, OsRickettsia = not_normalized_perSample$Rickettsia)

library(tidyverse)
not_normalized_perSample <- not_normalized_perSample %>%
  mutate(sex = ifelse(grepl("M$", Group), "Male", "Female"))

normalized_perSample <- normalized_perSample %>%
  mutate(sex = ifelse(grepl("M$", Group), "Male", "Female"))

#Box plot Actin normalized data with Tukey letters, on transformed scale
p1<-ggplot(normalized_perSample, aes(x=Group, y=bcRickettsia)) +
  geom_boxplot(show.legend = FALSE , aes(fill=sex)) + scale_fill_manual(values=c( "#bdbdbd", "#636363")) +
  geom_text(data = generate_label_df(normalized_perSample, tHSD, 'Group', 8), aes(x = plot.labels, y = V1, label = labels)) +
  ggtitle("Rickettsia", subtitle = paste("Actin normalized, transformed scale, Box Cox lambda = ", round(lambda, digits=3))) +
  xlab("Treatments") + ylab("Relative quantity (transformed)") +
  theme(axis.text = element_text(size = 11)) +
  theme(axis.title = element_text(size = 12)) +
  theme(plot.title = element_text(face="bold")) +
  scale_x_discrete(labels = str_wrap(c("Antibiotic female", "Antibiotic male",
```

```
  "Control female", "Control male", "Colony female", "Colony male"), width = 10))
p1
#ggsave(filename = "Rickettsia-AN-Ts.tiff", path="Paper/original scale", width = 600, height = 700, units="px", device='tiff', dpi=96)


#Box plot Actin normalized data with Tukey letters, on original scale
p2<-ggplot(normalized_perSample, aes(x=Group, y=OsRickettsia)) +
  geom_boxplot(show.legend = FALSE , aes(fill=sex)) + scale_fill_manual(values=c("#bdbdbd", "#636363")) +
  geom_text(data = generate_label_df(normalized_perSample, tHSD, 'Group', 9), aes(x = plot.labels, y = V1, label = labels)) +
  ggtitle("Rickettsia", subtitle = paste("Actin normalized, original scale, Box Cox lambda = ", round(lambda, digits=3))) +
  xlab("Treatments") + ylab("Relative quantity") +
  theme(axis.text = element_text(size = 11)) +
  theme(axis.title = element_text(size = 12)) +
  theme(plot.title = element_text(face="bold")) +
  scale_x_discrete(labels = str_wrap(c("Antibiotic female", "Antibiotic male", "Control female", "Control male", "Colony female", "Colony male"), width = 10))
p2
#ggsave(filename = "Rickettsia-AN-Os.tiff", path="Paper/original scale", width = 600, height = 700, units="px", device='tiff', dpi=96)


#Box plot NOT normalized data with Tukey letters, on transformed scale
p3<-ggplot(not_normalized_perSample, aes(x=Group, y=bcRickettsia)) +
  geom_boxplot(show.legend = FALSE , aes(fill=sex)) + scale_fill_manual(values=c("#bdbdbd", "#636363")) +
  geom_text(data = generate_label_df(not_normalized_perSample, tHSD_nn, 'Group', 8), aes(x = plot.labels, y = V1, label = labels)) +
  ggtitle("Rickettsia", subtitle = paste("Not normalized, transformed scale, Box Cox lambda = ", round(lambda_nn, digits=3))) +
  xlab("Treatments") + ylab("Relative quantity (transformed)") +
  theme(axis.text = element_text(size = 11)) +
  theme(axis.title = element_text(size = 12)) +
  theme(plot.title = element_text(face="bold")) +
  scale_x_discrete(labels = str_wrap(c("Antibiotic female", "Antibiotic male", "Control female", "Control male", "Colony female", "Colony male"), width = 10))
p3
#ggsave(filename = "Rickettsia-NN-Ts.tiff", path="Paper/original scale", width = 600, height = 700, units="px", device='tiff', dpi=96)


#Box plot NOT normalized data with Tukey letters, on original scale
p4<-ggplot(not_normalized_perSample, aes(x=Group, y=OsRickettsia)) +
  geom_boxplot(show.legend = FALSE , aes(fill=sex)) + scale_fill_manual(values=c("#bdbdbd", "#636363")) +
  geom_text(data = generate_label_df(not_normalized_perSample, tHSD_nn, 'Group', 9), aes(x = plot.labels, y = V1, label = labels)) +
  ggtitle("Rickettsia", subtitle = paste("Not normalized, original scale, Box Cox lambda = ", round(lambda_nn, digits=3))) +
  xlab("Treatments") + ylab("Relative quantity") +
  theme(axis.text = element_text(size = 11)) +
  theme(axis.title = element_text(size = 12)) +
  theme(plot.title = element_text(face="bold")) +
  scale_x_discrete(labels = str_wrap(c("Antibiotic female", "Antibiotic male", "Control female", "Control male", "Colony female", "Colony male"), width = 10))
p4
#ggsave(filename = "Rickettsia-NN-Os.tiff", path="Paper/original scale", width = 600, height = 700, units="px", device='tiff', dpi=96)


ggsave(filename = "Rickettsia-Ts.tiff", path="Paper/Graphs", arrangeGrob(p3, p1,nrow = 1), width = 322, height = 185, units="mm", device='tiff', dpi=300, compression = "lzw")
ggsave(filename = "Rickettsia-Os.tiff", path="Paper/Graphs", arrangeGrob(p4, p2,nrow = 1), width = 322, height = 185, units="mm", device='tiff', dpi=300, compression = "lzw")

############### End of the R Script for Rickettsia data analysis ###############
```

```r
################ Beginning of the R Script for Arsenophonus data analysis ################

library(ggplot2)
library(cowplot)
library(tidyr)
library(plyr)
library(multcompView)
library(MASS)
library(readr)
library(naniar)
library(gridExtra)
library(vcd)

## locate and read data
raw_data_sample <- read_csv("Data_samples.csv")
#View(raw_data_sample)
raw_data_curves <- read_csv("Data_curves.csv")
#View(raw_data_curves)

#Clean up the data
raw_data_sample$Group <- gsub('-','_', raw_data_sample$Group)
raw_data_sample <- subset(raw_data_sample, Group != "NC" & Group != "PC_D1" & Group != "PC_D2" & Group != "PC_D3")

raw_data_sample <- raw_data_sample %>% replace_with_na(replace = list(Arsenophonus= 40.00000))


#Function for plotting standard curves
ggplotRegression <- function (fit) {

  require(ggplot2)

  ggplot(fit$model, aes_string(x = names(fit$model)[2], y = names(fit$model)[1])) +
    geom_point() +
    stat_smooth(method = "lm", col = "red") +
    ggtitle(paste("Standard curve for", names(fit$model)[1]),
            subtitle = substitute(paste("Adj. R"^2," = ",r,
                                        ", Intercept = ",i,
                                        ", Slope = ",s,
                                        ", p = ",p),
                                        list(r= signif(summary(fit)$adj.r.squared, 5),i= signif(fit$coef[[1]],5 ),s=signif(fit$coef[[2]], 5), p=signif(summary(fit)$coef[2,4], 5))
                                       )
           ) + xlab(paste(names(fit$model)[2], "(log10)")) + ylab(paste(names(fit$model)[1], "(Ct)"))
}


#Make standard curves, save models to "curves" and parameters to "curves_param", and plot the curves
curves_param <- data.frame()
curves <- list()
raw_data_curves_trans <- raw_data_curves
raw_data_curves_trans[, "Quantity"] = log10(raw_data_curves_trans[, "Quantity"])
for(i in names(raw_data_curves[, -c(2, 3)])[-1]){
  curves[[i]] <- lm(paste(i, " ~ Quantity"), raw_data_curves_trans[, -c(2, 3)])
  tmp <- data.frame(lapply(curves[[i]][["coefficients"]][1:2], type.convert), stringsAsFactors=FALSE)
  tmp[, "rsquared"] = signif(summary(curves[[i]])$adj.r.squared, 5)
  tmp[, "p"] = signif(summary(curves[[i]])$coef[2,4], 5)
  names(tmp)[1] <- "intercept"
  names(tmp)[2] <- "slope"
  curves_param <-  rbind(curves_param, cbind(Gene = i, tmp))
  rm(tmp)
  print(ggplotRegression(curves[[i]]))
  }
rm(raw_data_curves_trans)

pcr_amount <- function(vec, a, b) {
  res <- 10 ^ ((vec - a)/b)
```

```
  return(res)
}

get_amounts <- function(df, intercept, slope) {

  amounts <- mapply(function(d, a, b) pcr_amount(d, a, b),
                    d = df, a = intercept, b = slope)
  amounts <- as.data.frame(amounts)

  return(amounts)
}


pcr_normalize <- function(vec, ref, mode) {
  if (mode == 'subtract') {
    res <- vec - ref
  } else if (mode == 'divide') {
    res <- vec / ref
  } else {
    stop("mode can be one of 'subtract' or 'divide'.")
  }
  return(res)
}


relative_quantities_perSample <- get_amounts(
  raw_data_sample[3:7],
  intercept = curves_param$intercept,
  slope = curves_param$slope
)

normalized_perSample <- pcr_normalize(relative_quantities_perSample, relative_quantities_perSample$Actin, mode = 'divide')
normalized_perSample <- cbind(normalized_perSample, raw_data_sample[c("ID", "Group")])

not_normalized_perSample <- relative_quantities_perSample
not_normalized_perSample <- cbind(not_normalized_perSample, raw_data_sample[c("ID", "Group")])

#Comment next line to apply normalization
#normalized_perSample <- not_normalized_perSample

powerTransform <- function(y, lambda1, lambda2 = NULL, method = "boxcox") {

  boxcoxTrans <- function(x, lam1, lam2 = NULL) {

    # if we set lambda2 to zero, it becomes the one parameter transformation
    lam2 <- ifelse(is.null(lam2), 0, lam2)

    if (lam1 == 0L) {
      log(y + lam2)
    } else {
      (((y + lam2)^lam1) - 1) / lam1
    }
  }

  switch(method
         , boxcox = boxcoxTrans(y, lambda1, lambda2)
         , tukey = y^lambda1
  )
}

# run the box-cox transformation to determine optimal lambda
bc <- boxcox(normalized_perSample$Arsenophonus ~ normalized_perSample$Group)
bc_nn <- boxcox(not_normalized_perSample$Arsenophonus ~ not_normalized_perSample$Group)

#save and print lambda
(lambda <- bc$x[which.max(bc$y)])
(lambda_nn <- bc_nn$x[which.max(bc_nn$y)])

#BoxCox transform the data
normalized_perSample <- mutate(normalized_perSample, bcArsenophonus = powerTransform(
```

```
normalized_perSample$Arsenophonus, lambda))
not_normalized_perSample <- mutate(not_normalized_perSample, bcArsenophonus = powerTransform(not_normalized_perSample$Arsenophonus, lambda_nn))

#ANOVA model using normalized, transformed data
ANOVA_model_norm <- aov(normalized_perSample$bcArsenophonus~Group, data= normalized_perSample)
par(mfrow = c(2, 2))
plot(ANOVA_model_norm)
summary(ANOVA_model_norm)
ANOVA_model_norm

#ANOVA model using NOT normalized, transformed data
ANOVA_model_nn <- aov(not_normalized_perSample$bcArsenophonus~Group, data= not_normalized_perSample)
par(mfrow = c(2, 2))
plot(ANOVA_model_nn)
summary(ANOVA_model_nn)
ANOVA_model_nn

#Post-hoc Tukey test
tHSD <- TukeyHSD(ANOVA_model_norm, ordered = FALSE, conf.level = 0.95)
tHSD
tHSD_nn <- TukeyHSD(ANOVA_model_nn, ordered = FALSE, conf.level = 0.95)
tHSD_nn

#Function to generate letter groupings for graphing
generate_label_df <- function(d, HSD, flev, colNo){
  # Extract labels and factor levels from Tukey post-hoc
  Tukey.levels <- HSD[[flev]][,4]
  Tukey.labels <- multcompLetters(Tukey.levels)['Letters']
  plot.labels <- names(Tukey.labels[['Letters']])

  # Get highest quantile for Tukey's 5 number summary and add a bit of space to buffer between
  # upper quantile and label placement
  names(d)[colNo] <- "transformed"
  boxplot.df <- ddply(d, flev, function (x) max(fivenum(x$transformed)) + 0.2)

  # Create a data frame out of the factor levels and Tukey's homogenous group letters
  plot.levels <- data.frame(plot.labels, labels = Tukey.labels[['Letters']],
                            stringsAsFactors = FALSE)

  # Merge it with the labels
  labels.df <- merge(plot.levels, boxplot.df, by.x = 'plot.labels', by.y = flev, sort = FALSE)

  return(labels.df)
}

normalized_perSample <- mutate(normalized_perSample, OsArsenophonus = normalized_perSample$Arsenophonus)
not_normalized_perSample <- mutate(not_normalized_perSample, OsArsenophonus = not_normalized_perSample$Arsenophonus)

library(tidyverse)
not_normalized_perSample <- not_normalized_perSample %>%
  mutate(sex = ifelse(grepl("M$", Group), "Male", "Female"))

normalized_perSample <- normalized_perSample %>%
  mutate(sex = ifelse(grepl("M$", Group), "Male", "Female"))

#Box plot Actin normalized data with Tukey letters, on transformed scale
p1<-ggplot(normalized_perSample, aes(x=Group, y=bcArsenophonus)) +
  geom_boxplot(show.legend = FALSE , aes(fill=sex)) + scale_fill_manual(values=c("#bdbdbd", "#636363")) +
  geom_text(data = generate_label_df(normalized_perSample, tHSD, 'Group', 8), aes(x = plot.labels, y = V1, label = labels)) +
  ggtitle("Arsenophonus", subtitle = paste("Actin normalized, transformed scale, Box Cox lambda = ", round(lambda, digits=3))) +
  xlab("Treatments") + ylab("Relative quantity (transformed)") +
  theme(axis.text = element_text(size = 11)) +
  theme(axis.title = element_text(size = 12)) +
```

```r
  theme(plot.title = element_text(face="bold")) +
  scale_x_discrete(labels = str_wrap(c("Antibiotic female", "Antibiotic male", "Control female", "Control male", "Colony female", "Colony male"), width = 10))
p1
#ggsave(filename = "Arsenophonus-AN-Ts.tiff", path="Paper/original scale", width = 600, height = 700, units="px", device='tiff', dpi=96)


#Box plot Actin normalized data with Tukey letters, on original scale
p2<-ggplot(normalized_perSample, aes(x=Group, y=OsArsenophonus)) +
  geom_boxplot(show.legend = FALSE , aes(fill=sex)) + scale_fill_manual(values=c("#bdbdbd", "#636363")) +
  geom_text(data = generate_label_df(normalized_perSample, tHSD, 'Group', 9), aes(x = plot.labels, y = V1, label = labels)) +
  ggtitle("Arsenophonus", subtitle = paste("Actin normalized, original scale, Box Cox lambda = ", round(lambda, digits=3))) +
  xlab("Treatments") + ylab("Relative quantity") +
  theme(axis.text = element_text(size = 11)) +
  theme(axis.title = element_text(size = 12)) +
  theme(plot.title = element_text(face="bold")) +
  scale_x_discrete(labels = str_wrap(c("Antibiotic female", "Antibiotic male", "Control female", "Control male", "Colony female", "Colony male"), width = 10))
p2
#ggsave(filename = "Arsenophonus-AN-Os.tiff", path="Paper/original scale", width = 600, height = 700, units="px", device='tiff', dpi=96)


#Box plot NOT normalized data with Tukey letters, on transformed scale
p3<-ggplot(not_normalized_perSample, aes(x=Group, y=bcArsenophonus)) +
  geom_boxplot(show.legend = FALSE , aes(fill=sex)) + scale_fill_manual(values=c("#bdbdbd", "#636363")) +
  geom_text(data = generate_label_df(not_normalized_perSample, tHSD_nn, 'Group', 8), aes(x = plot.labels, y = V1, label = labels)) +
  ggtitle("Arsenophonus", subtitle = paste("Not normalized, transformed scale, Box Cox lambda = ", round(lambda_nn, digits=3))) +
  xlab("Treatments") + ylab("Relative quantity (transformed)") +
  theme(axis.text = element_text(size = 11)) +
  theme(axis.title = element_text(size = 12)) +
  theme(plot.title = element_text(face="bold")) +
  scale_x_discrete(labels = str_wrap(c("Antibiotic female", "Antibiotic male", "Control female", "Control male", "Colony female", "Colony male"), width = 10))
p3
#ggsave(filename = "Arsenophonus-NN-Ts.tiff", path="Paper/original scale", width = 600, height = 700, units="px", device='tiff', dpi=96)


#Box plot NOT normalized data with Tukey letters, on original scale
p4<-ggplot(not_normalized_perSample, aes(x=Group, y=OsArsenophonus)) +
  geom_boxplot(show.legend = FALSE , aes(fill=sex)) + scale_fill_manual(values=c("#bdbdbd", "#636363")) +
  geom_text(data = generate_label_df(not_normalized_perSample, tHSD_nn, 'Group', 9), aes(x = plot.labels, y = V1, label = labels)) +
  ggtitle("Arsenophonus", subtitle = paste("Not normalized, original scale, Box Cox lambda = ", round(lambda_nn, digits=3))) +
  xlab("Treatments") + ylab("Relative quantity") +
  theme(axis.text = element_text(size = 11)) +
  theme(axis.title = element_text(size = 12)) +
  theme(plot.title = element_text(face="bold")) +
  scale_x_discrete(labels = str_wrap(c("Antibiotic female", "Antibiotic male", "Control female", "Control male", "Colony female", "Colony male"), width = 10))

p4
#ggsave(filename = "Arsenophonus-NN-Os.tiff", path="Paper/original scale", width = 600, height = 700, units="px", device='tiff', dpi=96)


ggsave(filename = "Arsenophonus-Ts.tiff", path="Paper/Graphs", arrangeGrob(p3, p1,nrow = 1), width = 322, height = 185, units="mm", device='tiff', dpi=300, compression = "lzw")
ggsave(filename = "Arsenophonus-Os.tiff", path="Paper/Graphs", arrangeGrob(p4, p2,nrow = 1), width = 322, height = 185, units="mm", device='tiff', dpi=300, compression = "lzw")
```

```r
write.csv2(not_normalized_perSample,'Not normalized per sampleA.csv')
write.csv2(normalized_perSample,'Normalized per sampleA.csv')


no<-aggregate(Arsenophonus ~ Group, data=normalized_perSample, function(x) {sum(is.na(x))}, na.action = NULL)
yes<-aggregate(cbind(count = Arsenophonus) ~ Group, data = normalized_perSample, FUN = function(x){NROW(x)})
Ars_counts <- cbind(yes, no[c("Arsenophonus")])
Ars_counts
names(Ars_counts)[1] <- "Treatment"
names(Ars_counts)[2] <- "Positive"
names(Ars_counts)[3] <- "Negative"
Ars_counts

#Sum up the totals, calculate percentage for easy interpretation and export
Ars_counts_export <- mutate(Ars_counts, Total = Ars_counts$Positive + Ars_counts$Negative)
Ars_counts_export <- mutate(Ars_counts_export, PercentNegative = Ars_counts_export$Negative / Ars_counts_export$Total)
write.csv2(Ars_counts_export,'Arsenophonus-presence.csv')

matrix.please<-function(x) {
  m<-as.matrix(x[,-1])
  rownames(m)<-x[,1]
  m
}

Ars_counts <- matrix.please(Ars_counts)
mosaic(Ars_counts, shade=TRUE, legend=TRUE, labeling= labeling_border(rot_labels = c(0,0,0,0),
                                                                      just_labels = c("center",
                                                                                      "center",
                                                                                      "center",
                                                                                      "right"), set_varnames = list(A = "", B = "qPCR result")))
library(rcompanion)
PT = pairwiseNominalIndependence(Ars_counts,
                                 fisher = TRUE,
                                 gtest  = FALSE,
                                 chisq  = FALSE,
                                 digits = 3,
                                 method='holm',
                                 cramer = 'TRUE',
                                 workspace=2e8)

PT
#write.csv2(PT,'Arsenophonus-presence-Fisher.csv')

cldList(comparison = PT$Comparison,
        p.value    = PT$p.adj.Fisher,
        threshold  = 0.05)

############### End of the R Script for Arsenophonus data analysis ###############
```
